# The immuno-behavioural covariation associated with the treatment response to bumetanide in young children with autism spectrum disorder

**DOI:** 10.1038/s41398-022-01987-x

**Published:** 2022-06-03

**Authors:** Qingyang Li, Lingli Zhang, Haidi Shan, Juehua Yu, Yuan Dai, Hua He, Wei-Guang Li, Christelle Langley, Barbara J. Sahakian, Yin Yao, Qiang Luo, Fei Li

**Affiliations:** 1 Department of Computational Biology, School of Life Sciences, Fudan University, 200438 Shanghai, China; 2 Department of Developmental and Behavioural Pediatric & Child Primary Care, Brain and Behavioural Research Unit of Shanghai Institute for Pediatric Research and MOE-Shanghai Key Laboratory for Children’s Environmental Health, Xinhua Hospital, Shanghai Jiao Tong University School of Medicine, 200092 Shanghai, China; 3 Center for Experimental Studies and Research, The First Affiliated Hospital of Kunming Medical University, 650032 Kunming, China; 4 Collaborative Innovation Center for Brain Science, Department of Anatomy and Physiology, Shanghai Jiao Tong University School of Medicine, 200025 Shanghai, China; 5 Department of Psychiatry and the Behavioural and Clinical Neuroscience Institute, University of Cambridge, Cambridge, CB21TN UK; 6 National Clinical Research Center for Aging and Medicine at Huashan Hospital, State Key Laboratory of Medical Neurobiology and Ministry of Education Frontiers Center for Brain Science, Institutes of Brain Science and Institute of Science and Technology for Brain-Inspired Intelligence, Fudan University, 200433 Shanghai, China; 7 Human Phenome Institute, Fudan University, 201203 Shanghai, China; 8 Center for Computational Psychiatry, Ministry of Education-Key Laboratory of Computational Neuroscience and Brain-Inspired, Research Institute of Intelligent Complex Systems, Fudan University, 200040 Shanghai, China

**Keywords:** Predictive markers, Autism spectrum disorders

## Abstract

Bumetanide, a drug being studied in autism spectrum disorder (ASD) may act to restore gamma-aminobutyric acid (GABA) function, which may be modulated by the immune system. However, the interaction between bumetanide and the immune system remains unclear. Seventy-nine children with ASD were analysed from a longitudinal sample for a 3-month treatment of bumetanide. The covariation between symptom improvements and cytokine changes was calculated and validated by sparse canonical correlation analysis. Response patterns to bumetanide were revealed by clustering analysis. Five classifiers were used to test whether including the baseline information of cytokines could improve the prediction of the response patterns using an independent test sample. An immuno-behavioural covariation was identified between symptom improvements in the Childhood Autism Rating Scale (CARS) and the cytokine changes among interferon (IFN)-*γ*, monokine induced by gamma interferon and IFN-*α*2. Using this covariation, three groups with distinct response patterns to bumetanide were detected, including the best (21.5%, *n* = 17; Hedge’s g of improvement in CARS = 2.16), the least (22.8%, *n* = 18; *g* = 1.02) and the medium (55.7%, *n* = 44; *g* = 1.42) responding groups. Including the cytokine levels significantly improved the prediction of the best responding group before treatment (the best area under the curve, AUC = 0.832) compared with the model without the cytokine levels (95% confidence interval of the improvement in AUC was [0.287, 0.319]). Cytokine measurements can help in identifying possible responders to bumetanide in ASD children, suggesting that immune responses may interact with the mechanism of action of bumetanide to enhance the GABA function in ASD.

## Introduction

Autism spectrum disorder (ASD) affects about 1% children around the world [1] and can cause lifelong disability and elevate premature mortality [2]. Currently, no medication that can cure ASD or all of its core symptoms is available [1]. The recent success of repurposing drugs for novel treatments in psychiatry has been highlighted [3], with one of the examples given being the use of bumetanide to improve the core symptoms in ASD [4–8]. The most frequent adverse events were hypokalemia, increased urine elimination, loss of appetite, dehydration and asthenia. The heterogeneity in the treatment effect of bumetanide among ASD patients was significant, ranging from 37.3% to 47.62% in the randomised clinical trials (RCTs) in China [5, 8] and 51.80% [6] or from 26.3% to 45.2% [7] in the RCTs in France. Besides, there were also studies reported nonsignificant treatment effect of bumetanide for patients with ASD [9]. Understanding this heterogeneity is essential for its clinical applicability and requires further investigation of its underlying mechanism of action to achieve precision medicine for ASD children.

The use of bumetanide as a potential drug to improve symptoms in ASD is based on a hypothesised pathoetiology of ASD, namely the delayed developmental switch of the gamma-aminobutyric acid (GABA) functioning from excitatory to inhibitory [10–12]. In the valproate and fragile X rodent models of autism, this GABA-switch can be facilitated by the reduction of intracellular chloride concentration, which is mediated by a sequential expression of the main chloride transporters, such as the potassium (K)-Cl co-transporters 2 (KCC2) and the importer Na-K-Cl cotransporter 1 (NKCC1) [12]. Therefore, bumetanide as an NKCC1 inhibitor has been tested for its ability to restore GABA function in ASD [5–7, 13, 14]. However, these transporters can also be influenced by other molecules, such as cytokines, which are a number of small cell-signalling proteins closely interacting with each other to modulate the immune reactions. The cytokines have been implicated not only in brain development [15], but also in GABAergic transmission [16–18]. It has been reported that the interferon (IFN)-*γ* can decrease the levels of NKCC1 and the α-subunit of Na^+^-K^+^-ATPase, contributing to the restore of inhibitory GABA function [16]. In mice subjected to maternal deprivation, the interleukin (IL)-1 has also been found to reduce the expression of KCC2, delaying the developmental switch of the GABA function and thereby possibly contributing to the pathophysiology of developmental disorders such as ASD [17, 18]. Therefore, a question naturally arises that whether the treatment effect of bumetanide for ASD can be affected by the immune responses in the patients.

Indeed, compared with healthy controls, changes of the cytokine levels have already been reported in patients with ASD [19–22]. Recent meta-analyses showed that the levels of anti-inflammatory cytokines IL-10 and IL-1 receptor antagonist (Ra) were decreased [20], while proinflammatory cytokines IL-1*β*, IL-6 and anti-inflammatory cytokines IL-4, IL-13 were elevated in blood of patients with ASD [21]. The levels of IFN-*γ*, IL-6, tumour necrosis factor (TNF)-*α*, granulocyte-macrophage colony-stimulating factor (GM-CSF) and IL-8 were observed to be elevated [22] in postmortem brain tissues of ASD patients, and increased level of IFN-*γ*, monocyte chemotactic protein (MCP)-1, IL-8, leukaemia inhibitory factor (LIF) and interferon-gamma inducible protein (IP)-10 were found in another study [23]. These widely spread changes suggest that the cytokine signalling in ASD may be better characterised by multivariate patterns of cytokines. In literatures, many associations had been reported between the levels of cytokines (e.g., MCP-1, IL-1*β*, IL-4, IL-6, etc.) and both core symptoms and adaptive functions in children with ASD [24–26]. Therefore, it has been suggested that cytokines may be used as biomarkers to identify different subsets within ASD. In each of these subsets the patients with ASD may share a commonly immune-related pathoetiology and therefore may have similar profiles of response to treatment [27]. Based on these previous findings, we analysed data acquired through the Shanghai Xinhua ASD registry, China, that began in 2016 to test the hypothesis that the immune activity of patients might help to identify the best responders to bumetanide in ASD.

## Materials and methods

### Participants

The ASD participants were recruited from the Shanghai Xinhua ASD registry at Shanghai Jiaotong University Medical School Affiliated Xinhua Hospital in Shanghai, China, including the participants from two previous registered clinical studies, i.e., CHICtr-OPC-16008336 and NCT03156153. The patients were diagnosed with ASD according to the Diagnostic and Statistical Manual of Mental Disorders, Fifth Edition (DSM-5). Diagnoses were confirmed with the Autism Diagnostic Observation Schedule (ADOS), and a Children Autism Rating Scale (CARS) total score of no less than 30. Exclusion criteria include liver and kidney dysfunction; a history of allergy to sulfa drugs; abnormal electrocardiography; genetic or chromosomal abnormalities; suffering from nervous system diseases (e.g., epilepsy, etc.). Comprehensive behavioural assessments and collections of clinical samples were performed for all patients. Between May 1st, 2018, to April 30th, 2019, a total of 90 ASD children, aged 3–10 years old, under a 3-month stable treatment of bumetanide without behavioural interventions and any concomitant psychoactive medications had both blood draws and behavioural assessments. Among these patients, 11 of them were further excluded due to the lack of the follow-up data at month 3. A group of 37 children, under 3-month stable treatment of placebo without behavioural interventions and any concomitant psychoactive medications had both blood draws and behavioural assessments. Therefore, the current analysis used a subsample of 116 young children with ASD, whose blood samples were available both before and after the treatment. The blood samples were sent in three batches (Discovery Set: *n* = 37 on December 4, 2019; Validation Set: *n* = 42 on May 22, 2019; and Control Set: *n* = 37 on January 5, 2022) to measure the serum levels of 48 cytokines for the immune response (Table S1), and the clinical symptoms were assessed using CARS, ADOS and the Social Responsiveness Scale (SRS).

Following the protocols of previous studies [8], bumetanide treatment consisted of two 0.5 mg tablets per day for three months, given at 8:00 a.m. and 4:00 p.m. The tablet size is 8 mm diameter x 2 mm thickness, which is quite small. Each time, the patient took half of a tablet, which was not difficult for most of the patients. However, the careers were recommended to grind the half-tablet into powder and give the powder in water, if necessary. Possible side effects were closely monitored during the treatment. Blood parameters (serum potassium and uric acid) were monitored via laboratory tests (Table S2) and symptoms (thirst, diuresis, nausea, vomiting, diarrhoea, constipation, rash, palpitation, headache, dizziness, shortness of breath, and any other self-reported symptoms) were telephone interviewed (Table S3), and both of them were reported to the research team by telephone at 1 week and 1 month after the initiation of treatment and at the end of the treatment period. The cytokine levels of the children with gastrointestinal problems were compared with those without such problems (Table S4). Behavioural assessments of CARS and ADOS and measurements of cytokine levels were performed at the baseline before the treatment and after the 3-month treatment. The behavioural assessment of SRS was used at the baseline only. The study was conducted in accordance with the provisions of the Declaration of Helsinki and Good Clinical Practice guidelines and was approved by the Ethics Committee of Xinhua Hospital affiliated to Shanghai Jiao Tong University School of Medicine. Written informed consent was obtained from the parent or legal guardian of each participant before sample collection.

### Measures

#### Clinical assessments

The CARS was used to diagnose and evaluate the severity of clinical symptoms of ASD patients. The CARS consisted of 15 items rated on a 7-point scale from one to four; higher scores are associated with a higher level of impairment. Total scores can range from a low of 15 to a high of 60; scores below 30 indicate that the individual is in the non-autistic range, scores between 30 and 36.5 indicate mild to moderate autism, and scores from 37 to 60 indicate severe autism. We further categorised these items into three subscales [28]: Social impairment, Negative emotionality and Distorted sensory response. ADOS was used as a supplement to gauge disease severity, and it contained total score items and four modules for assessment of Social interaction, Communication, Play, and Imaginative use of materials for individuals suspected of having ASD [29]. SRS identified a wide spectrum of deficits in reciprocal social behaviour, ranging from absent to severe, based on observations of a child’s behaviour in naturalistic social settings, focused on the behaviour of a child or adolescent between the ages of 4 and 18 years. It was a 65-item questionnaire that is completed by teacher, a parent, and/or another adult caregiver. Scoring is on a four-point Likert Scale. Five subscales are also provided: Social Awareness (AWA), Social cognition (COG), Social Communication (COM), Social Motivation (MOT), and Autistic Mannerism (MANN) [30].

#### Cytokine levels

For each subject peripheral blood was collected, centrifuged at 2300 rpm for 10 min, and the plasma harvested. Plasma was aliquoted and stored at −70 °C until cytokine analysis. A plane of 48 cytokines, chemokines, and growth factors were measured using the Bio-Plex multiple immunoassays (Bio‐Plex®; BIO‐RAD Laboratories, Inc.). Prior to setting up our assays, the Bio-Plex 200 System plane Reader instrument was calibrated according to manufacturer instructions. To prepared experimental samples, frozen plasma aliquots were passively thawed to room temperature and diluted four-fold in assay buffer (15 μL sample + 45 μL sample diluent HB). After preparation of capture bead mixture, and standards, the immunoassay was carried out on a 96-well plane. The experimental steps were in accordance with the instructions. Data acquisition was set to a 50-bead count minimum per analyte per well. Unknown sample cytokine concentrations were processed and presented with Bio-plex Manager software using a standard curve derived from the known reference cytokine concentrations supplied by the manufacturer. A five-parameter model was used to calculate final concentrations and values were expressed in pg/ml. The sensitivity of this assay allowed the detection of cytokine concentrations within the following ranges: IFN-*α*2 3.6-3992.4 pg/ml; IFN-*γ* 0.9-14556.8 pg/ml; IL-1*β* 0.3-5375.0 pg/ml; IL-4 0.2-3455.0 pg/ml; IL-6 0.4-5961.8 pg/ml; IL-8 0.2-15570.4 pg/ml; MIG 5.7-30955.2 pg-ml; TNF-*α* 3.3-52256.0 pg/ml, etc. Concentrations obtained below the sensitivity limit of detection (LOD) of the method were excluded from all subsequent analyses.

### Statistical analysis

#### Data preprocessing

After excluding the cytokines whose values were less than the limit of detection, 35 cytokines were included in our analysis. Data from three batches, containing both the baseline and the change (the difference value from the baseline and the follow-up data) of the cytokine levels, were min-max normalised and log transformed separately. To adjust for the batch effect, we applied an empirical Bayes approach to the baseline of cytokine levels using the ‘ComBat’ parametric algorithm provided in the R-package ‘sva’ [31]. Principle component analysis (PCA) was used to visualise the non-biological variation due to the batch effect and was repeated to confirm its adjustment (Fig. S1). Before and after treatment, we compared the demographic parameters (i.e., sex proportion, age, body mass index [BMI]) and symptom severity (i.e., ADOS and CARS) between these three data sets using two-sided Mann–Whitney *U*-test, or Pearson’s chi-squared test where applicable.

#### Multivariate association analysis to characterise the immuno-behavioural covariation

First, the pairwise correlation between the CARS_total score and each of the 35 cytokine levels were assessed by the Spearman-rank correlation. The correlation between the change in the CARS_total score and the change in each of the 35 cytokine levels after the treatment was also tested. The false-discovery rate (FDR) was used to correct for the multiple comparisons.

Second, to uncover the multivariate association between the behavioural assessments and the cytokine levels, we employed the sparse canonical correlation analysis (sCCA) provided in a R-package ‘sRDA’ (version 1.0.0) [32]. Canonical correlation analysis (CCA) is a classical method for determining the relationship between two sets of variables. Given two data sets **X**_1_ and **X**_2_ of dimensions *n* × *p*_1_ and *n* × *p*_2_, respectively, from *n* observations, CCA seeks the pairs of linear combinations (i.e., the canonical variables), one from the variables in **X**_1_ and the other from the variables in **X**_2_, that are maximally correlated with each other. However, some variables may make negligible but non-zero contributions to the canonical variables. sCCA was developed to address this issue. sCCA applies an L_1_ penalty to the canonical weights, which forces some of them to take a value of exactly zero. Mathematically,

$$\begin{array}{ll}\mathop {{\max }}\limits_{w_1,w_2} w_1^T{{{\mathbf{X}}}}_1^T{{{\mathbf{X}}}}_2w_2\;{{{\mathrm{subject}}}}\;{{{\mathrm{to}}}}\left\| {w_1} \right\|^2 = 1,\left\| {w_2} \right\|^2\\ = 1,\left\| {w_1} \right\|_1 \ll {{{\boldsymbol{c}}}}_1,\left\| {w_2} \right\|_1 \le {{{\boldsymbol{c}}}}_2,w_1 \in R^{{{{\boldsymbol{p}}}}_1},w_2 \in R^{{{{\boldsymbol{p}}}}_2}.\end{array}$$

Here, *c*_1_ and *c*_2_ are assumed to fall within the bounds $1 \le c_1 \ll \sqrt {p_1}$ and $1 \le c_2 \ll \sqrt {p_2}$, where *p*_1_ and *p*_2_ are the numbers of features in **X**_1_ and **X**_2_, respectively. We refer to ***w***_1_ and ***w***_2_ as the canonical weights, and **X**_1_***w***_1_ and **X**_2_***w***_2_ as the canonical scores. Therefore, this algorithm could identify a linear combination of three CARS subscales (i.e., the behavioural-component) that was significantly associated with another linear combination of a few cytokine levels (i.e., the cytokine-component). Meanwhile, the sparsity of this algorithm ensured only the key cytokines driving the behavioural association were selected in the immune component.

In the Discovery Set, we explored the sCCA between CARS subscales and cytokine levels using the baseline data or using the changes between baseline and follow-up. The significance of an identified canonical correlation was assessed by 5000 permutations [32]. Only the significant canonical components were retained. The Validation Set was used to confirm these significant canonical components among patients with bumetanide treatment. The Control Set was used to test whether the association between the cytokine-component and the behavioural-component was significant in the placebo group. Sensitivity analysis was conducted using the data from the Validation Set by re-evaluating the canonical correlation after controlling for the potential confounders, including age, sex, BMI and the canonical variables at the baseline. Using the cytokine-component score (*x*-axis) and the behavioural-component score (*y*-axis) established by sCCA, each patient could be mapped onto a two-dimensional, called the immuno-behavioural covariation plane, characterising the immuno-behavioural covariation in the response patterns to the bumetanide treatment among young children with ASD.

#### Clustering analysis to identify the immuno-behavioural groups

We applied *K*-means, an unsupervised clustering algorithm, to identify the clusters of patients according to the immuno-behavioural covariation. The patients in each cluster (i.e., an immuno-behavioural group within ASD) had the similar canonical scores, suggesting they shared the similar patterns of response to bumetanide in both immune system and clinical behaviour. The cluster structures were first identified using the Discovery Set and then validated using the Validation Set. The optimal number of clusters was selected based on the elbow (maximum change) of the scree plot using the Hubert statistic implemented in the R-package ‘NBclust’ [33].

To demonstrate the distinct patterns of response to bumetanide in the immuno-behavioural groups, we applied the one-sample *t*-test to the after-treatment changes of both the CARS subscales and the cytokines selected by the sCCA algorithm as described above. We also compared these changes among the above identified immuno-behavioural groups using the Kruskal–Wallis rank sum test with the FDR correction for multiple comparisons.

#### Prediction of the treatment response to bumetanide using the baseline information

To predict the response to bumetanide of a patient with ASD, we trained the classifiers for the immuno-behavioural groups identified above using the baseline information before the treatment. The baseline information included the 35 cytokine levels, 3 types of clinical assessment (CARS, SRS, ADOS) and 2 demographic parameters (sex and age). The classifiers included the Oblique Random Forest (ORF), Partial Least Squares (PLS), sparse Linear Discriminant Analysis (sLDA), Neural Networks (NN) and Support Vector Machine (SVM) as implemented in the R-package ‘caret’ with both feature selection and oversampling [34]. The models were first trained using the Discovery Set, and their performances were compared using the Validation Set. The 95% confidence interval of the difference between the areas under the curves of a pair of models was constructed by 100 bootstraps.

First, we tested whether including the cytokine levels at the baseline could improve the prediction of the immuno-behaviourally defined responders. The averaged performances of these five classifiers (i.e., the averaged area under the curve, $\overline {{{{\mathrm{AUC}}}}}$) were reported and compared.

Second, we tested whether the behaviourally defined responders were more difficult to be predicted at the baseline compared with the immuno-behaviourally defined ones. In the previous clinical trials of bumetanide for ASD [5–7], the proportion of patients who responded positively to the treatment was between 30% and 40%. Therefore, we divided the patients with ASD into two groups according to the ΔCARS_total (= the baseline CARS_total – the follow-up CARS_total) with a cutoff of 2.5 (*n* = 22, 27.85% of the patients had ΔCARS_total >2.5) or 2 (*n* = 32, 40.51% of the patients had ΔCARS_total >2) points.

## Results

### Participants

Three data sets of children with ASD (*n* = 116) were used in the current study. Discovery Set (*n* = 37) had a mean age of 47 months (±17.35 months), 18.92% of whom were girls; Validation Set (*n* = 42) had a mean age of 54 months (±20.19 months), 23.81% of whom were girls; Control Set (*n* = 37) had a mean age of 50 months (±10.72 months), 13.16% of whom were girls. No significant difference in clinical characteristics or cytokine levels was identified between these three data sets (Table 1 and Table S5).

**Table 1:** The demographic and clinical (mean(SD)) characteristics of three data sets.

	Discovery Set (*n* = 37)	Validation Set (*n* = 42)	Control Set (*n* = 37)	Dis. vs. Val.		Dis. vs. Con.		Val. vs. Con.	
				Statistic (df)^a^	*P*-value	Statistic (df)^a^	*P*-value	Statistic (df)^a^	*P*-value
Age, months, mean (sd)	47.08 (17.35)	53.57 (20.19)	49.64 (10.72)	905	0.212	795	0.332	823	0.813
Female sex, *n* (%)	7 (18.92)	10 (23.81)	5 (13.16)	0.06 (1)	0.800	0.13 (1)	0.715	0.87 (1)	0.351
BMI, mean (sd)	17.80 (5.02)	16.48 (3.54)	16.41 (1.54)	672	0.338	653	0.886	591	0.099
Baseline									
CARS									
CARS_total	37.28 (3.50)	37.98 (4.13)	38.76 (5.12)	817	0.701	757	0.570	756	0.685
CARS_S	24.73 (2.59)	25.31 (2.86)	26.16 (3.70)	836	0.568	836	0.161	703	0.362
CARS_N	7.18 (0.82)	7.12 (1.02)	7.26 (1.31)	730	0.639	684	0.842	779	0.853
CARS_D	7.36 (0.86)	7.65 (1.05)	7.46 (1.05)	903	0.213	688	0.876	896	0.342
ADOS^b^									
ADOS_S	6.95 (3.70)	6.29 (2.53)	6.26 (1.59)	762	0.885	721	0.854	773	0.806
ADOS_C	9.46 (2.82)	10.14 (2.86)	10.68 (2.29)	875	0.336	879	0.061	728	0.498
ADOS_P	2.30 (1.51)	2.33 (1.59)	2.55 (1.22)	781	0.976	760	0.541	725	0.471
ADOS_I	2.11 (2.84)	2.14 (1.98)	2.08 (1.46)	869	0.358	801	0.292	787	0.917
SRS^c^									
SRS_AWA	9.83 (4.92)	10.17 (4.57)	10.67 (3.38)	503	0.793	209	0.979	393	0.821
SRS_COG	15.57 (6.82)	16.62 (6.57)	17.50 (4.12)	522	0.596	237	0.436	357	0.740
SRS_COM	26.52 (12.55)	29.69 (12.55)	31.06 (7.69)	558	0.309	253	0.231	365	0.834
SRS_MOT	12.43 (5.49)	13.88 (6.52)	15.39 (4.50)	545	0.402	269	0.108	330	0.438
SRS_MANN	10.13 (6.72)	14.17 (7.91)	15.72 (3.95)	615	0.071	321	0.279	308	0.257
SRS_total	74.48 (32.25)	84.40 (34.37)	90.33 (18.40)	566	0.257	266	0.127	346	0.611
Baseline—follow-up									
ΔCARS									
ΔCARS_total	1.54 (1.40)	1.90 (1.34)	0.89 (1.43)	885	0.288	543	0.088	1090	0.005
ΔCARS_S	0.92 (1.10)	1.24 (0.95)	0.54 (1.08)	920	0.156	573	0.165	1083	0.005
ΔCARS_N	0.41 (0.58)	0.30 (0.56)	0.14 (0.72)	700	0.425	556	0.104	892	0.348
ΔCARS_D	0.30 (0.49)	0.49 (0.51)	0.21 (0.59)	927	0.122	624	0.380	1024	0.024

*BMI* body mass index, *CARS* Childhood Autism Rating Scale, *ADOS* the Autism Diagnostic Observation Schedule, *SRS* the Social Responsiveness Scale, *CARS_total* CARS total score, *CARS_S* CARS score on social impairment domain, *CARS_N* CARS score on negative emotionality domain, *CARS_D* CARS score on distorted sensory response domain, *ADOS_S* ADOS score on social interaction, *ADOS_C* ADOS score on communication, *ADOS_P* ADOS score on play, *ADOS_I* ADOS score on imaginative use of materials, *SRS_AWA* SRS score on social awareness, *SRS_COG* SRS score on social cognition, *SRS_COM* SRS score on social communication, *SRS_MOT* SRS score on social motivation, *SRS_MANN* SRS score on autistic mannerism, *SRS_total* SRS total score.

^a^Mann–Whitney *U*-test for non-normal features, while chi-square test for sex.

^b^Sample size for ADOS data in Discovery Set, Validation Set and Control Set are 36, 41 and 36.

^c^Sample size for SRS data in Discovery Set, Validation Set and Control Set are 22, 41 and 17.

### Changes after the administration of bumetanide

Seventy-nine patients were treated with bumetanide for 3 months, and the CARS total score decreased after the treatment (effect size Cohen’s d = 1.26, *t*_*78*_ = 11.21, *p* < 0.001). The treatment effect showed no difference between the Discovery Set and the Validation Set (ΔCARS_total: mean(±SD): 1.54 (±1.40) vs. 1.90 (±1.34)). Consistent to the previous studies of the low-dose bumetanide for ASD, the side effects were rarely reported (Tables S2 and S3). No significant difference in the cytokine levels between the children with and without the gastrointestinal problems at the baseline (Table S4). A number of cytokine levels were changed significantly after the treatment of bumetanide, but none of them was changed significantly after the treatment of placebo (Table S6). No significant pairwise association could be identified in the Discovery Set, the Validation Set and the Control Set among four groups of variables, including the baseline CARS total score, the baseline cytokine levels, the change of CARS total score, and the changes of cytokine levels (Fig. S2).

### Covariation between symptom improvement and cytokine changes

**Fig. 1:**
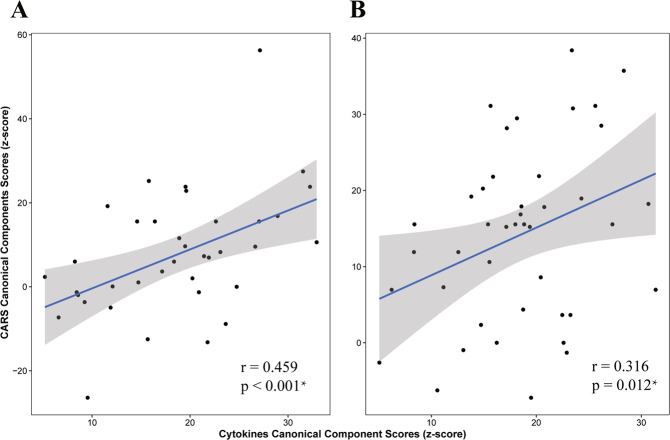
Sparse canonical correlation analysis. We carried out sparse canonical correlation analysis in **A** Discovery Set and **B** Validation Set. The canonical scores between CARS and cytokines, which were min-max normalised and log transformed, were significant related in both data sets.

Using the Discovery Set, we found a canonical correlation (*r* = 0.459; *p* < 0.001 by permutation; Fig. 1A) between 2 canonical components (i.e., cytokine-component and behavioural-component) by sCCA. The cytokine-component was a combination of the changes of the three cytokine levels, including the MIG, IFN-*α*2, IFN-*γ*. The behavioural-component was a combination of the changes of three subscales of the CARS scores, including the social impairment score, negative emotionality score, distorted sensory response score. Applying these canonical weights to an independent data set (i.e., the Validation Set), we confirmed the correlation between the cytokine-component and the behavioural-component (*r* = 0.316; *p* = 0.012 by permutation; Fig. 1B). In contrast, applying these canonical weights to the Control Set, we found that the association between cytokine-component and behavioural-component was not significant (*r* = 0.038, *p* = 0.821), which might indicate that the association between cytokine-component and behavioural-component was related to the drug effect.

*Sensitivity analysis* Using the Validation Set, we found that the correlation between the canonical components identified above remained significant (Table S7). At the baseline, we also found that the cytokines canonical scores were associated with other clinical assessments, including the SRS_total (*r* = −0.269; *t*_63_ = −2.21, *p* = 0.031), ADOS_S (*r* = 0.296; *t*_76 _= 2.70, *p* = 0.009), ADOS_P (*r* = −0.244; *t*_76_ = −2.19, *p* = 0.032), and ADOS_I (*r* = −0.251; *t*_76_ = −2.26, *p* = 0.027).

### Three distinct response patterns revealed by the immuno-behavioural covariation

**Fig. 2:**
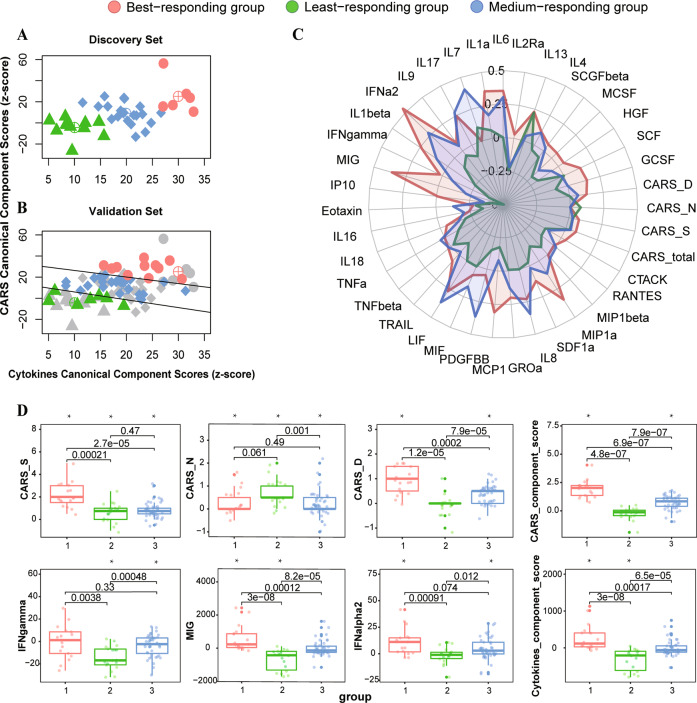
Differences in three immuno-behavioural groups. *K*-means cluster plot on the immuno-behavioural plane. *K*-means cluster analysis was carried out in Discovery Set (**A**) and the patients from the Validation Set were mapped to this immuno-behavioural plane (**B**). **C** Radar chart for the ratios of changes to baseline of CARS and cytokine levels in 3 immuno-behavioural groups. **D** Boxplot for the significant changes of CARS and cytokine levels in 3 immuno-behavioural groups.

Using both the CARS- and the cytokine- component scores, we found that the patients fell into three clusters (Fig. 2A, B and Fig. S3). No significant difference in the distribution of patients among these three clusters between the Discovery Set and the Validation Set. Besides, no significant difference in the baseline of CARS scores and cytokine levels among these 3 clusters. Comparing among these three groups of patients in both data sets (Fig. 2C, D and Table 2; Fig. S4), we found that the best responding group (*n* = 17) had the greatest reduction in the CARS total score (ΔCARS_total: 3.32(±1.47), Hedge’s *g* = 2.16, *p* < 0.001), which was most prominent in the social impairment score (ΔCARS_S: 2.15(±1.13), *g* = 1.81, *p* < 0.001) and increased cytokine levels (ΔMIG: *g* = 0.72, *p* = 0.012; ΔIFN-*α*2: *g* = 0.84, *p* = 0.006). The least responding group (*n* = 18) had the least reduction in the CARS total score (ΔCARS_total: 1.03(±0.96), *g* = 1.02, *p* < 0.001), which was most significant in the negative emotionality score (ΔCARS_N: 0.69(±0.55), *g* = 1.21, *p* < 0.001), and decreased in the least responding group (ΔMIG: *g* = −1.07, *p* < 0.001; ΔIFN-*γ*: *g* = −1.32, *p* < 0.001). The medium responding group had a significant decrease in both the CARS_total score and all subscales with a small effect size each, while the IFN-*γ* level decreased and the IFN-*α*2 level increased in this group (Table 2).

**Table 2:** Changes in CARS and cytokine levels after the treatment in three immuno-behavioural groups.

	Best responding group (*n* = 17)				Least responding group (*n* = 18)				Medium responding group (*n* = 44)				$\chi _2^2$	*p*^d^
	mean(SD)	*t*^a^	Hedge’s *g*	*p*^d^	mean(SD)	*t*^b^	Hedge’s *g*	*p*^d^	mean (SD)	*t*^c^	Hedge’s *g*	*p*^d^		
CARS_total	3.32 (1.47)	9.34	2.16	<0.001	1.03 (0.96)	4.53	1.02	<0.001	1.41 (0.97)	9.61	1.42	<0.001	25.69	<0.001
CARS_S	2.15 (1.13)	7.84	1.81	<0.001	0.64 (0.87)	3.11	0.70	0.010	0.86 (0.76)	7.56	1.14	<0.001	20.35	<0.001
CARS_N	0.35 (0.58)	2.51	0.58	0.026	0.69 (0.55)	5.40	1.21	<0.001	0.20 (0.52)	2.61	0.39	0.016	10.63	0.005
CARS_D	0.91 (0.51)	7.41	1.71	<0.001	−0.06 (0.42)	−0.57	−0.13	0.579	0.39 (0.34)	7.57	1.14	<0.001	30.33	<0.001
IFN-γ	−0.37 (14.72)	−0.10	−0.02	0.927	−14.50 (10.46)	−5.88	−1.32	<0.001	−3.83 (9.54)	−2.66	−0.39	0.016	13.81	0.002
IFN-α2	11.15 (12.60)	3.65	0.84	0.006	−1.63 (7.87)	−0.88	−0.20	0.502	4.68 (10.00)	3.10	0.46	0.008	12.46	0.002
MIG	586.62 (777.11)	3.11	0.72	0.012	−698.83 (623.10)	−4.76	−1.07	<0.001	−6.53 (470.77)	−0.09	−0.01	0.927	31.68	<0.001

*CARS_total* CARS total score, *CARS_S* CARS score on social impairment domain, *CARS_N* CARS score on negative emotionality domain, *CARS_D* CARS score on distorted sensory response domain, *IFN-γ* Interferon gamma, *IFN-α2* interferon alpha 2, *MIG* monokine induced by gamma interferon.

^a^The degree of freedom for the One-sample *t*-test statistic is 16.

^b^The degree of freedom for the One-sample *t*-test statistic is 17.

^c^The degree of freedom for the One-sample *t*-test statistic is 43.

^d^FDR correction for multiple comparisons.

### Baseline cytokine levels helped in identifying the treatment-responders

**Fig. 3:**
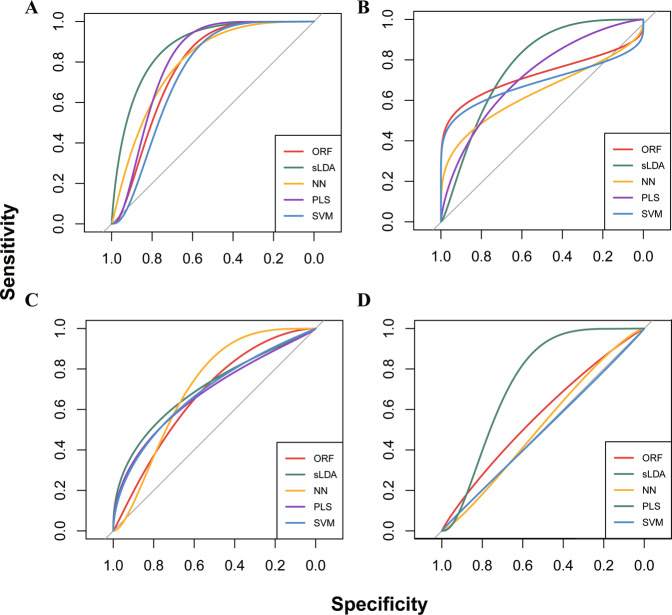
ROC curve for prediction for the immuno-behaviourally defined responding group. The classifiers included the Oblique Random Forest (ORF) model, Partial Least Squares (PLS) model, Support Vector Machine (SVM) model, sparse Linear Discriminant Analysis (sLDA) model and Neural Networks (NN) model. Based on the immuno-behavioural covariation plane, the models were trained to predict the response to bumetanide for the children with ASD. As described in the main text, the models were trained using the Discovery Set, and tested using the Validation Set. The performances of the classification accuracy in the testing data set were reported in this figure. **A** Models with the cytokine levels at the baseline for predicting patients with ASD in the best responding group. **B** Models without the cytokine levels at the baseline for predicting patients with ASD in the best responding group. **C** Models with the cytokine levels at the baseline for predicting patients with ASD in the least responding group. **D** Models without the cytokine levels at the baseline for predicting patients with ASD in the least responding group.

Training five different types of classifiers with Discovery Set and testing the performance using Validation Set, we found that including the cytokine levels at the baseline significantly improved the prediction accuracy for both the best responding group ($\overline {{{{\mathrm{AUC}}}}}$ with/without the baseline cytokine levels = 0.768/0.646; improvement in $\overline {{{{\mathrm{AUC}}}}} = {{{\mathrm{0}}}}{{{\mathrm{.122}}}}$, 95% confidence interval (CI): (0.103,0.130); Fig. 3A, B) and the least responding group ($\overline {{{{\mathrm{AUC}}}}}$ with/without the baseline cytokine levels = 0.698/0.618; improvement in $\overline {{{{\mathrm{AUC}}}}} = {{{\mathrm{0}}}}{{{\mathrm{.080}}}}$, 95%CI: (0.064,0.097); Fig. 3C, D). Each of the five models showed a better performance after including the cytokine levels into the model (Table S8). Furthermore, we found that the behaviourally defined responders according to the ΔCARS_total with a cutoff of 2.5 were more difficult to be predicted at the baseline ($\overline {{{{\mathrm{AUC}}}}} = {{{\mathrm{0}}}}{{{\mathrm{.569}}}}$) compared with the immuno-behaviourally defined responders ($\overline {{{{\mathrm{AUC}}}}} = {{{\mathrm{0}}}}{{{\mathrm{.768}}}}$; Table S9; Fig. S5A). Similar results were found when using the threshold of 2 to behaviourally define the responders (Fig. S5B).

## Discussion

In this study, we observed a significant improvement of clinical symptoms with bumetanide treatment in children with ASD, and such improvement was associated with a pattern of changes in three cytokine levels, namely the IFN-*γ*, MIG and IFN-*α*2 (*r* = 0.459 in the Discovery Set and *r* = 0.316 in the Validation Set). These cytokine levels at the baseline could improve the prediction of the bumetanide responders compared with using the behavioural assessments alone, and the best predictor achieved an AUC of 0.83 in the independent test data set (Table S8). The implications of these findings may be twofold: (1) a significant part of the clinical heterogeneity in the treatment effect of bumetanide for ASD is associated with the differences in the immune system of patients, and (2) the component score of cytokines had a potential to construct a blood signature for predicting and monitoring the bumetanide treatment in young children with ASD.

Our finding of an immuno-behavioural covariation highlights the role of the immune system in the clinical effect of bumetanide in young children with ASD. IFN-*γ*, as a T helper cell 1 (Th1) cytokine with proinflammatory effects, was selected by the sCCA algorithm to be one of the three cytokines to form the canonical score that was associated with the improvement in CARS. Compared to controls, higher level of IFN-*γ* has been reported in the brain tissue [22], cerebrospinal fluid (CSF) [23], plasma [35] and peripheral blood mononuclear cell (PBMC) [36] in ASD patients, and lower level has been observed in neonatal dried blood samples (n-DBSS) of ASD children [37]. Accumulating evidences support that IFN-*γ* can inhibit chloride secretion [38] and down-regulate both the NKCC1 expression [16, 38] and the Na^+^-K^+^-ATPase expression [16], which had been implicated in the GABAergic dysfunction in ASD [10, 39]. Indirectly, an animal study also showed that stimulation with high concentration of IFN-*γ* could increase the expression of IL-1*β* [40], which is an inflammatory cytokine that can affect the expression of chloride transporters and delay the developmental switch of GABA signalling [17]. Therefore, the immune system may interact with the mechanism of action of bumetanide to restore the GABA function in ASD.

The cytokine-symptom association was identified in the changes after the treatment of bumetanide but not before the treatment, suggesting that bumetanide might interact with the cytokines and the changes of which contributed to the treatment effect of bumetanide. Animal studies showed a rapid brain efflux of bumetanide, but a number of clinical trials have shown a significant treatment effect for neuropsychiatric disorders, including ASD, epilepsy and depression [41, 42]. These findings may suggest the possible systemic effects of bumetanide as a neuromodulator for these neuropsychiatric disorders. Considering its molecular structure, bumetanide has been recently identified by an in vitro screen of small molecules that can act as an anti-proinflammatory drug via interleukin inhibition [43]. This anti-proinflammatory activity of bumetanide might alter the blood levels of cytokines outside the brain-blood-barrier (BBB). In fact, it has already been reported that bumetanide reduced the Lipopolysaccharide-induced production of proinflammatory cytokines following a direct pulmonary administration in RAW264.7 cells and in lung-injured mice [44]. These inflammatory signalling messengers may pass the BBB [45] and influence the neuronal chloride homoeostasis via, for example, altering the KCC2 expression [18]. The plausibility of reducing inflammation to enhance the KCC2 expression has recently been discussed in a 2020 review [17]. Indeed, we found that the best responding group (Hedge’s *g* = 2.16 for the reduction of CARS total score) had the greatest increase in the cytokine-component score. In the contrary, the least responding group (*g* = 1.02) had the greatest decrease in the cytokine-component score. Taken together, these findings suggest that bumetanide may be a drug to inhibit NKCC1 and enhance KCC2 through its interplay with the cytokines inside and/or outside of the brain.

It was disappointing that the phase 3 clinical studies assessing bumetanide in the treatment of ASD were terminated after the middle analysis, which showed no sign of effectiveness, led by two pharmaceutical companies Servier and Neurochlore [https://servier.com/en/communique/servier-and-neurochlore-announce-the-main-results-of-the-two-phase-3-clinical-studies-assessing-bumetanide-in-the-treatment-of-autism-spectrum-disorders-in-children-and-adolescents/]. To date, the detailed results of these Phase 3 trials have not been published, we could not learn more details about the operation and the outcomes. Considering the nature of heterogeneity in the aetiology and pathology of ASD, the development of biological first-line treatment may only be effective in certain subtypes [46]. Therefore, an important direction of bumetanide treatment is to identify potential biomarkers for a high responsiveness to this drug, and thereby to identify those patients who are more likely to benefit from this treatment. Indeed, our findings of an association between cytokine levels and the symptom improvement after the bumetanide treatment demonstrated the potential of cytokine levels as blood biomarkers for precision medicine in ASD. Our findings may suggest that the identified canonical score of cytokines had a potential to construct a blood signature for predicting and monitoring the bumetanide treatment in young children with ASD. Accurately identifying patients who are likely to respond positively to bumetanide can facilitate the precision medicine for ASD. Our prediction model based on the cytokine levels before the treatment may provide a potentially new tool for the precision medicine of ASD. Given the inherit heterogeneity of ASD, it is of great clinical value to accurately identify the subgroup of ASD patients that are likely to respond positively to its medical treatments [47]. Multiple factors, including both genetic and environmental factors, could contribute to the heterogeneity of ASD and its response to treatment [12, 19, 48]. For example, prenatal insults including maternal infection and subsequent immunological activation during gestation may increase the risk of autism in the child [19]. Increased exposure to air pollution during gestation was also associated with abnormalities in mitochondrial metabolism during childhood, which may also increase the risk for ASD [47]. However, our findings suggested that using the cytokine levels improved the prediction of response to the bumetanide treatment for ASD in three folds: (1) The immuno-behavioural covariation enabled the identification of more homogenous subgroup of ASD in terms of response to bumetanide. Using five models and an independent test data set, we demonstrated consistent evidence that the responding group identified by the immuno-behavioural covariation could be significantly better predicted by the baseline information compared with the responder group defined by the CARS alone. (2) Combining the cytokine levels with clinical assessments of CARS, ADOS and SRS before treatment, we achieved a higher accuracy of 84.3% in identifying the ASD children that were likely to respond positively to the bumetanide treatment. (3) The blood cytokine levels are more easily accessible in clinical practice.

There are several limitations of our study. Although we had two separate data sets to validate our findings, the sample sizes were limited. Previously a sex difference in the cytokine-symptom association had been reported, but we could not test such sex difference owing to the small numbers of girls with ASD in our sample [49]. Hence, future multi-centre, prospective studies with lager sample sizes are necessary to confirm the current findings. Second, the hypothesised molecular mechanism underlying the bumetanide treatment effect for ASD requires causal confirmation in animal studies.

In summary, we identified an association between the changes of the cytokine levels and the improvements in symptoms after the bumetanide treatment in young children with ASD, and found that the treatment effect of bumetanide can be better characterised by an immuno-behavioural covariation. This finding may provide new clinically important evidence supporting the hypothesis that immune responses may interact with the mechanism of bumetanide to restore the GABAergic function in ASD. This finding may also have relevance for determining enriched samples of ASD children to participate in novel drug treatment studies of drugs with a similar mode of action to bumetanide, but with potentially greater efficacy and fewer side effects.

## Supplementary information

Supplementary Materials

## Data Availability

The data of this study are available under reasonable and ethically approved request to the corresponding authors.

## Code availability

The R code of this study is available at https://github.com/qluo2018/ImmunoASD4BTN.
