# Supplementary material for: The immuno-behavioural covariation associated with the treatment response to bumetanide in young children with autism spectrum disorder
Source: Transl Psychiatry. 2022 Jun 3;12:228. doi: 10.1038/s41398-022-01987-x (PMC9166783; doi:10.1038/s41398-022-01987-x)
Supplement: Supplementary file 1 — Supplementary Materials [file 41398_2022_1987_MOESM1_ESM.docx]

Supplementary Materials

**The immuno-behavioural covariation associated with the treatment response to bumetanide in young children with autism spectrum disorder**

Li, Zhang, Shan, *et al.*

Table of Contents

[Table S1. Measured immune factors, clinical assessments and demographic parameters 2](#_Toc98341366)

[Table S2. Side effects measured by blood parameters reported during the treatment. 3](#_Toc98341367)

[Table S3. Side effects measured by symptoms reported during the treatment. 5](#_Toc98342091)

[Table S4. The cytokine levels of the children with gastrointestinal problems were compared with those without such problems. 7](#_Toc98342894)

[Table S5. The baseline levels of cytokines in three data sets. 8](#_Toc98342895)

[Table S6. The change levels of cytokines in three data sets. 9](#_Toc98342896)

[Table S7. Correlation between the identified canonical components after controlling for baseline variables. 11](#_Toc98342898)

[Table S8. Comparison of AUC between the models including and without including the cytokines to predict the immuno-behaviourally defined best responding group and the least responding group. 12](#_Toc98342899)

[Table S9. Comparison of AUC between the models predicting the immuno-behaviourally defined responder group and the behaviourally defined responder group 13](#_Toc98342900)

[Figure S1. Adjustment for batch effects. 14](#_Toc98342901)

[Figure S2. Pairwise association between CARS total score and cytokine. 15](#_Toc98342902)

[Figure S3. The scree plot for selection of the optimal number of clusters. 16](#_Toc98342903)

[Figure S4. Boxplot for the changes of CARS and cytokine levels in 3 immuno-behavioural groups. 17](#_Toc98342904)

[Figure S5. ROC curve for the prediction of treatment response defined by CARS. 20](#_Toc98342910)

**Supplementary Tables and Figures**

**Table S1. Measured immune factors, clinical assessments and demographic parameters**

| **Abbr.** | **Description** | **Abbr.** | **Description** | **Abbr.** | **Description** |
| --- | --- | --- | --- | --- | --- |
| IL-1α | Interleukin 1 alpha | CCL2 | Monocyte chemotactic protein 1 (MCP-1) | MIF | Macrophage migration inhibitory factor |
| IL-1β | Interleukin 1 beta | CCL3 | Macrophage inflammatory protein 1-α (MIP-1α) | CARS_total | CARS total score |
| IL-2Ra | Interleukin 2 receptor alpha | CCL4 | Macrophage inflammatory protein 1-β (MIP-1β) | CARS_S | CARS score on social impairment domain |
| IL-4 | Interleukin 4 | CCL5 | Regulated on Activation Normal T-cell Expressed and Secreted (RANTES) | CARS_N | CARS score on negative emotionality domain |
| IL-6 | Interleukin 6 | CCL11 | Eotaxin | CARS_D | CARS score on distorted sensory response domain |
| IL-7 | Interleukin 7 | CCL27 | Cutaneous T cell attracting cytokine (CTACK) | ADOS_S | ADOS score on social interaction |
| IL-8 | Interleukin 8 (CXCL8) | CXCL1 | GRO alpha oncogene (GROα) | ADOS_C | ADOS score on communication |
| IL-9 | Interleukin 9 | CXCL9 | Monokine induced by gamma interferon (MIG) | ADOS_P | ADOS score on play |
| IL-13 | Interleukin 13 | CXCL10 | Interferon gamma-induced protein 10 (IP10) | ADOS_I | ADOS score on imaginative use of materials |
| IL-16 | Interleukin 16 | CXCL12 | Stromal cell-derived factor 1 alpha (SDF-1α) | SRS_AWA | SRS score on social awareness |
| IL-17 | Interleukin 17 | G-CSF | Granulocyte colony stimulating factor (C17orf33) | SRS_COG | SRS score on social cognition |
| IL-18 | Interleukin 18 | M-CSF | Macrophage colony stimulating factor | SRS_COM | SRS score on social communication |
| IFN-α2 | Interferon alpha 2 | PDGF-β | Platelet derived growth factor beta-beta homodimer | SRS_MOT | SRS score on social motivation |
| IFN-γ | Interferon gamma | HGF | Hepatocyte growth factor | SRS_MANN | SRS score on autistic mannerism |
| TNF-α | Tumor necrosis factor alpha | LIF | Leukemia inhibitory factor | SRS_total | SRS total score |
| TNF-β | Tumor necrosis factor beta | SCF | Stem cell factor | BMI | Body mass index |
| TRAIL | TNF-related apoptosis-inducing ligand | SCGF-β | Stem cell growth factor beta | Age/Sex |  |

**Table S2. Side effects measured by blood parameters reported during the treatment*.**

| id | before-K | before-U | 1-week-K | 1-week-U | 1-mon-K | 1-mon-U | 3-mon-K | 3-mon-U |
| --- | --- | --- | --- | --- | --- | --- | --- | --- |
| 434 | 0 | 0 | 0 | 0 | 0 | 0 | 0 | 0 |
| 448 | 0 | 0 | 0 | 0 | 0 | 0 | 0 | 0 |
| 449 | 0 | 0 | 0 | 0 | 0 | 0 | 1 | 0 |
| 463 | 0 | 0 | 0 | 0 | 0 | 0 | 0 | 0 |
| 509 | 0 | 0 | 0 | 0 | 0 | 0 | 0 | 0 |
| 535 | 0 | 0 | 0 | 0 | 0 | 0 | 0 | 0 |
| 559 | 0 | 0 | 0 | 0 | 0 | 0 | 0 | 0 |
| 566 | 0 | 0 | 0 | 0 | 0 | 0 | 1 | 0 |
| 587 | 0 | 0 | 0 | 0 | 0 | 0 | 0 | 0 |
| 619 | 0 | 0 | 0 | 0 | 0 | 0 | 0 | 0 |
| 621 | 0 | 0 | 0 | 0 | 0 | 0 | 0 | 0 |
| 628 | 0 | 0 | 0 | 0 | 0 | 0 | 0 | 0 |
| 631 | 0 | 0 | 0 | 0 | 0 | 0 | 0 | 0 |
| 633 | 0 | 0 | 0 | 0 | 0 | 0 | 0 | 0 |
| 673 | 0 | 0 | 0 | 0 | 0 | 0 | 0 | 0 |
| 675 | 0 | 0 | 0 | 0 | 0 | 0 | 0 | 0 |
| 687 | 0 | 0 | 0 | 0 | 0 | 0 | 0 | 0 |
| 695 | 0 | 0 | 0 | 0 | 0 | 0 | 0 | 0 |
| 774 | 0 | 0 | 0 | 0 | 0 | 0 | 0 | 0 |
| 785 | 0 | 0 | 0 | 0 | 0 | 0 | 0 | 0 |
| 803 | 0 | 0 | 0 | 0 | 0 | 0 | 0 | 0 |
| 810 | 0 | 0 | 0 | 0 | 0 | 0 | 1 | 0 |
| 833 | 0 | 0 | 0 | 1 | 0 | 0 | 0 | 1 |
| 838 | 0 | 0 | 0 | 0 | 0 | 0 | 0 | 0 |
| 850 | 0 | 0 | 0 | 0 | 0 | 0 | 0 | 0 |
| 858 | 0 | 0 | 0 | 0 | 0 | 0 | 0 | 0 |
| 863 | 0 | 0 | 0 | 0 | 0 | 0 | 0 | 0 |
| 875 | 0 | 0 | 0 | 0 | 0 | 0 | 0 | 0 |
| 877 | 0 | 0 | 0 | 0 | 0 | 0 | 0 | 0 |
| 884 | 0 | 0 | 0 | 0 | 0 | 0 | 0 | 0 |
| 894 | 0 | 0 | 0 | 0 | 0 | 0 | 0 | 0 |
| 899 | 0 | 0 | 0 | 0 | 0 | 0 | 0 | 0 |
| 909 | 0 | 0 | 0 | 0 | 0 | 0 | 0 | 0 |
| 914 | 0 | 0 | 0 | 0 | 0 | 0 | 0 | 0 |
| 916 | 0 | 0 | 0 | 0 | 0 | 0 | 0 | 0 |
| 927 | 0 | 0 | 0 | 1 | 0 | 0 | 0 | 0 |
| 929 | 0 | 0 | 0 | 0 | 0 | 0 | 0 | 0 |
| 938 | 0 | 0 | 0 | 0 | 0 | 0 | 0 | 0 |
| 941 | 0 | 0 | 0 | 0 | 0 | 0 | 1 | 0 |
| 942 | 0 | 0 | 0 | 0 | 0 | 0 | 1 | 1 |
| 963 | 0 | 0 | 0 | 0 | 0 | 0 | 0 | 0 |

**Table S2 (continued).**

| 964 | 0 | 0 | 0 | 0 | 0 | 0 | 0 | 0 |
| --- | --- | --- | --- | --- | --- | --- | --- | --- |
| 968 | 0 | 0 | 0 | 0 | 0 | 0 | 0 | 0 |
| 970 | 0 | 0 | 0 | 0 | 0 | 0 | 0 | 0 |
| 974 | 0 | 0 | 0 | 0 | 0 | 0 | 0 | 0 |
| 976 | 0 | 0 | 0 | 0 | 0 | 0 | 0 | 0 |
| 982 | 0 | 0 | 0 | 0 | 0 | 0 | 0 | 0 |
| 983 | 0 | 0 | 0 | 0 | 0 | 0 | 0 | 0 |
| 986 | 0 | 0 | 0 | 0 | 0 | 0 | 0 | 0 |
| 995 | 0 | 0 | 0 | 0 | 0 | 0 | 0 | 0 |
| 1013 | 0 | 0 | 0 | 0 | 0 | 0 | 0 | 0 |
| 1017 | 0 | 0 | 0 | 0 | 0 | 0 | 0 | 0 |
| 1026 | 0 | 0 | 0 | 0 | 0 | 0 | 0 | 0 |
| 1027 | 0 | 0 | 0 | 0 | 0 | 0 | 0 | 0 |
| 1029 | 0 | 0 | 0 | 0 | 0 | 0 | 0 | 0 |
| 1030 | 0 | 0 | 0 | 0 | 0 | 0 | 0 | 0 |
| 1033 | 0 | 0 | 0 | 0 | 0 | 0 | 0 | 0 |
| 1037 | 0 | 0 | 0 | 0 | 0 | 0 | 0 | 0 |
| 1039 | 0 | 0 | 0 | 0 | 0 | 0 | 0 | 0 |
| 1060 | 0 | 0 | 0 | 0 | 0 | 0 | 0 | 0 |
| 1067 | 0 | 0 | 0 | 0 | 0 | 0 | 0 | 0 |
| 1070 | 0 | 0 | 0 | 0 | 0 | 0 | 0 | 0 |
| 1090 | 0 | 0 | 0 | 0 | 0 | 0 | 0 | 0 |
| 1093 | 0 | 0 | 0 | 0 | 0 | 0 | 0 | 0 |
| 1094 | 0 | 0 | 0 | 0 | 0 | 0 | 0 | 0 |
| 1095 | 0 | 0 | 0 | 0 | 0 | 0 | 0 | 0 |
| 1096 | 0 | 0 | 0 | 0 | 0 | 0 | 0 | 0 |
| 1098 | 0 | 0 | 0 | 0 | 0 | 0 | 0 | 0 |
| 1106 | 0 | 0 | 0 | 0 | 0 | 0 | 0 | 0 |
| 1131 | 0 | 0 | 0 | 0 | 0 | 0 | 0 | 1 |
| 1141 | 0 | 0 | 0 | 0 | 0 | 0 | 0 | 0 |
| 1155 | 0 | 0 | 0 | 0 | 0 | 0 | 0 | 0 |
| 1157 | 0 | 0 | 0 | 0 | 0 | 1 | 0 | 0 |
| 1168 | 0 | 0 | 0 | 0 | 0 | 0 | 0 | 0 |
| 1226 | 0 | 0 | 0 | 0 | 0 | 0 | 0 | 0 |
| 1280 | 0 | 0 | 0 | 0 | 0 | 0 | 0 | 0 |
| 1283 | 0 | 0 | 0 | 1 | 0 | 0 | 0 | 0 |
| 1286 | 0 | 0 | 0 | 0 | 0 | 0 | 0 | 0 |
| 1330 | 0 | 0 | 0 | 0 | 0 | 0 | 0 | 0 |

*****Score = 0 means no symptoms; Score = 1 in K means hypokalemia (< 3.5 mmol/L); Score = 1 in U means increased urine elimination (> 417 umol/L).

before, at the baseline; 1-week, after 1-week treatment of bumetanide; 1-month, after 1-month treatment of bumetanide; 3-month, after 3-month treatment of bumetanide.

**Table S3. Side effects measured by symptoms reported during the treatment*.**

| **id** | **thirst** | | **diuresis** | **constipation** | **loss_of_appetite** | **nausea** | **vomiting** | **palpitation** | **diarrhoea** | **trouble_sleeping** |
| --- | --- | --- | --- | --- | --- | --- | --- | --- | --- | --- |
| 434 | 0 | 0 | | 0 | 0 | 0 | 0 | 0 | 0 | 0 |
| 448 | 0 | 0 | | 0 | 0 | 0 | 0 | 0 | 0 | 0 |
| 449 | 0 | 0 | | 0 | 0 | 0 | 0 | 0 | 0 | 0 |
| 463 | 0 | 0 | | 0 | 0 | 0 | 0 | 0 | 0 | 0 |
| 509 | 1 | 1 | | 0 | 0 | 0 | 0 | 0 | 0 | 0 |
| 535 | 0 | 0 | | 0 | 0 | 0 | 0 | 0 | 0 | 0 |
| 559 | 1 | 1 | | 0 | 0 | 0 | 0 | 0 | 0 | 0 |
| 566 | 1 | 1 | | 0 | 0 | 0 | 0 | 0 | 0 | 0 |
| 587 | 1 | 1 | | 0 | 0 | 0 | 0 | 0 | 0 | 0 |
| 619 | 1 | 1 | | 0 | 0 | 0 | 0 | 0 | 0 | 0 |
| 621 | 0 | 1 | | 0 | 0 | 0 | 0 | 0 | 0 | 0 |
| 628 | 1 | 1 | | 0 | 0 | 0 | 0 | 0 | 0 | 0 |
| 631 | 0 | 0 | | 0 | 0 | 0 | 0 | 0 | 0 | 0 |
| 633 | 0 | 0 | | 0 | 0 | 0 | 0 | 0 | 0 | 0 |
| 673 | 1 | 1 | | 1 | 0 | 0 | 0 | 0 | 0 | 0 |
| 675 | 0 | 0 | | 0 | 0 | 0 | 0 | 0 | 0 | 0 |
| 687 | 1 | 1 | | 0 | 0 | 0 | 0 | 0 | 0 | 0 |
| 695 | 0 | 0 | | 0 | 0 | 0 | 0 | 0 | 0 | 0 |
| 774 | 0 | 0 | | 0 | 0 | 0 | 0 | 0 | 0 | 0 |
| 785 | 0 | 0 | | 0 | 0 | 0 | 0 | 0 | 0 | 0 |
| 803 | 0 | 0 | | 0 | 0 | 0 | 0 | 0 | 0 | 0 |
| 810 | 0 | 0 | | 0 | 0 | 0 | 0 | 0 | 0 | 0 |
| 833 | 0 | 2 | | 0 | 0 | 0 | 0 | 0 | 0 | 0 |
| 838 | 0 | 0 | | 0 | 0 | 0 | 0 | 0 | 0 | 0 |
| 850 | 0 | 1 | | 0 | 0 | 0 | 0 | 0 | 0 | 0 |
| 858 | 0 | 0 | | 0 | 0 | 0 | 0 | 0 | 0 | 0 |
| 863 | 0 | 0 | | 0 | 0 | 0 | 0 | 0 | 0 | 0 |
| 875 | 0 | 1 | | 0 | 0 | 0 | 1 | 0 | 0 | 0 |
| 877 | 0 | 0 | | 0 | 0 | 0 | 0 | 0 | 0 | 0 |
| 884 | 0 | 1 | | 0 | 0 | 0 | 0 | 0 | 0 | 0 |
| 894 | 1 | 1 | | 0 | 0 | 0 | 0 | 0 | 0 | 0 |
| 899 | 1 | 1 | | 0 | 0 | 0 | 0 | 0 | 0 | 0 |
| 909 | 0 | 0 | | 0 | 0 | 0 | 0 | 0 | 0 | 0 |
| 914 | 0 | 0 | | 0 | 0 | 0 | 0 | 0 | 0 | 0 |
| 916 | 0 | 0 | | 1 | 0 | 0 | 0 | 0 | 0 | 0 |
| 927 | 0 | 1 | | 0 | 0 | 0 | 0 | 0 | 0 | 0 |
| 929 | 1 | 3 | | 0 | 0 | 0 | 0 | 0 | 0 | 0 |
| 938 | 1 | 1 | | 0 | 0 | 0 | 0 | 0 | 0 | 0 |
| 941 | 0 | 0 | | 0 | 0 | 0 | 0 | 0 | 0 | 0 |
| 942 | 0 | 0 | | 0 | 0 | 0 | 0 | 0 | 0 | 0 |
| 963 | 1 | 1 | | 0 | 0 | 0 | 0 | 0 | 0 | 0 |

**Table S3 (continued).**

| 964 | 0 | 0 | 0 | 0 | 0 | 0 | 0 | 0 | 0 |
| --- | --- | --- | --- | --- | --- | --- | --- | --- | --- |
| 968 | 0 | 0 | 0 | 0 | 0 | 0 | 0 | 0 | 0 |
| 970 | 0 | 0 | 0 | 0 | 0 | 0 | 0 | 0 | 0 |
| 974 | 0 | 0 | 0 | 0 | 0 | 0 | 0 | 0 | 0 |
| 976 | 0 | 1 | 0 | 0 | 0 | 0 | 0 | 0 | 0 |
| 982 | 0 | 0 | 0 | 0 | 0 | 0 | 0 | 0 | 0 |
| 983 | 1 | 1 | 0 | 0 | 0 | 0 | 0 | 0 | 0 |
| 986 | 0 | 0 | 0 | 0 | 0 | 0 | 0 | 0 | 0 |
| 995 | 1 | 1 | 0 | 0 | 0 | 0 | 0 | 0 | 0 |
| 1013 | 1 | 1 | 0 | 0 | 0 | 0 | 0 | 0 | 0 |
| 1017 | 0 | 0 | 0 | 0 | 0 | 0 | 0 | 0 | 0 |
| 1026 | 0 | 0 | 0 | 0 | 0 | 0 | 0 | 0 | 0 |
| 1027 | 0 | 1 | 0 | 0 | 0 | 0 | 0 | 0 | 0 |
| 1029 | 1 | 1 | 0 | 0 | 0 | 0 | 0 | 0 | 0 |
| 1030 | 0 | 2 | 0 | 0 | 0 | 0 | 0 | 0 | 0 |
| 1033 | 0 | 0 | 0 | 0 | 0 | 0 | 0 | 0 | 0 |
| 1037 | 0 | 1 | 0 | 0 | 0 | 0 | 0 | 0 | 0 |
| 1039 | 0 | 1 | 0 | 0 | 0 | 0 | 0 | 0 | 0 |
| 1060 | 0 | 1 | 0 | 1 | 0 | 0 | 0 | 0 | 0 |
| 1067 | 0 | 2 | 0 | 0 | 0 | 0 | 0 | 0 | 0 |
| 1070 | 0 | 1 | 0 | 0 | 0 | 0 | 0 | 0 | 0 |
| 1090 | 0 | 1 | 0 | 0 | 0 | 0 | 0 | 0 | 0 |
| 1093 | 0 | 1 | 0 | 0 | 0 | 0 | 0 | 0 | 0 |
| 1094 | 0 | 2 | 0 | 0 | 0 | 0 | 0 | 0 | 0 |
| 1095 | 0 | 1 | 0 | 0 | 0 | 0 | 0 | 0 | 0 |
| 1096 | 0 | 0 | 1 | 0 | 0 | 0 | 0 | 0 | 0 |
| 1098 | 1 | 1 | 0 | 0 | 0 | 0 | 0 | 0 | 0 |
| 1106 | 0 | 1 | 0 | 0 | 0 | 0 | 0 | 0 | 0 |
| 1131 | 0 | 1 | 0 | 0 | 0 | 0 | 0 | 0 | 0 |
| 1141 | 0 | 0 | 0 | 0 | 0 | 0 | 0 | 0 | 0 |
| 1155 | 0 | 0 | 0 | 0 | 0 | 0 | 0 | 0 | 0 |
| 1157 | 0 | 0 | 0 | 0 | 0 | 0 | 0 | 0 | 0 |
| 1168 | 0 | 1 | 0 | 0 | 0 | 0 | 0 | 0 | 0 |
| 1226 | 1 | 1 | 0 | 0 | 0 | 0 | 0 | 0 | 0 |
| 1280 | 0 | 0 | 0 | 0 | 0 | 0 | 0 | 1 | 0 |
| 1283 | 0 | 1 | 1 | 0 | 0 | 0 | 0 | 0 | 0 |
| 1286 | 0 | 0 | 0 | 0 | 0 | 0 | 0 | 0 | 0 |
| 1330 | 0 | 0 | 0 | 0 | 0 | 0 | 0 | 0 | 0 |

*****Score = 0, 1, 2 and 3 means none, mild, moderate and severe symptoms.

**Table S4. The cytokine levels of the children with gastrointestinal problems were compared with those without such problems.**

| Cytokines ^1^ | Children without gastrointestinal problems  (n=63) | Children with gastrointestinal problems  (n=15) | statistic ^2^ | P value ^3^ |
| --- | --- | --- | --- | --- |
| IL-1β | 0.38(0.24) | 0.40(0.23) | 360 | 0.447 |
| IL-6 | 0.52(0.24) | 0.52(0.24) | 307 | 0.234 |
| IL-8 | 0.40(0.24) | 0.40(0.23) | 241 | 0.059 |
| IFN-γ | 0.47(0.26) | 0.47(0.25) | 544 | 0.684 |
| TNF-α | 0.39(0.29) | 0.41(0.26) | 459 | 0.960 |
| MCP-1 | 0.39(0.22) | 0.40(0.23) | 437 | 0.870 |
| Eotaxin | 0.52(0.20) | 0.51(0.22) | 573 | 0.478 |
| IL-17 | 0.54(0.22) | 0.54(0.22) | 316 | 0.234 |
| IL-4 | 0.44(0.27) | 0.45(0.25) | 364 | 0.452 |
| IL-2Rα | 0.50(0.25) | 0.50(0.24) | 662 | 0.157 |
| MIG | 0.29(0.28) | 0.30(0.24) | 467 | 0.960 |
| MIP-1β | 0.46(0.23) | 0.46(0.24) | 466 | 0.960 |
| IFN-α2 | 0.53(0.25) | 0.54(0.24) | 344 | 0.395 |
| SDF-1α | 0.48(0.21) | 0.47(0.23) | 416 | 0.727 |
| IL-16 | 0.24(0.23) | 0.23(0.24) | 312 | 0.234 |
| LIF | 0.41(0.26) | 0.41(0.25) | 329 | 0.304 |
| TNF-β | 0.72(0.24) | 0.70(0.24) | 465 | 0.960 |
| MIF | 0.33(0.25) | 0.34(0.25) | 451 | 0.939 |
| RANTES | 0.45(0.23) | 0.45(0.22) | 493 | 0.939 |
| IL-18 | 0.35(0.23) | 0.36(0.22) | 579 | 0.452 |
| PDGF-β | 0.37(0.26) | 0.36(0.25) | 468 | 0.960 |
| IP-10 | 0.29(0.23) | 0.29(0.25) | 509 | 0.870 |
| IL-13 | 0.42(0.24) | 0.42(0.25) | 286 | 0.157 |
| MIP-1α | 0.41(0.28) | 0.43(0.27) | 406 | 0.698 |
| G-CSF | 0.40(0.24) | 0.42(0.24) | 222 | 0.052 |
| GROα | 0.66(0.28) | 0.63(0.25) | 439 | 0.870 |
| HGF | 0.43(0.27) | 0.44(0.26) | 516 | 0.860 |
| IL-1α | 0.53(0.24) | 0.52(0.24) | 409 | 0.699 |
| SCF | 0.44(0.26) | 0.44(0.25) | 396 | 0.645 |
| TRAIL | 0.41(0.24) | 0.41(0.22) | 534 | 0.699 |
| M-CSF | 0.36(0.21) | 0.37(0.21) | 568 | 0.480 |
| CTACK | 0.48(0.24) | 0.47(0.24) | 590 | 0.439 |
| IL-7 | 0.50(0.26) | 0.50(0.26) | 348 | 0.404 |
| IL-9 | 0.43(0.25) | 0.43(0.24) | 378 | 0.480 |
| SCGF-β | 0.59(0.23) | 0.58(0.23) | 504 | 0.874 |

^1^ Data [i.e., mean (SD)] were first normalized and second corrected for batch effect.

^2^ Mann-Whitney U test.

^3^ FDR adjustment for multiple testing.

**Table S5. The baseline levels of cytokines in three data sets.**

| Cytokines ^1^ | Discovery Set  (n=37) | Validation Set  (n=42) | Control Set  (n=37) | $\boldsymbol{\chi}_{\boldsymbol{2}}^{\boldsymbol{2}}$ | P value |
| --- | --- | --- | --- | --- | --- |
| IL-1β | 0.38(0.24) | 0.4(0.23) | 0.38(0.23) | 2.24 | 0.327 |
| IL-6 | 0.52(0.24) | 0.52(0.24) | 0.5(0.22) | 1.64 | 0.440 |
| IL-8 | 0.4(0.24) | 0.4(0.23) | 0.38(0.22) | 2.74 | 0.254 |
| IFN-γ | 0.47(0.26) | 0.47(0.25) | 0.47(0.27) | 0.00 | 0.999 |
| TNF-α | 0.39(0.29) | 0.41(0.26) | 0.41(0.29) | 0.41 | 0.813 |
| MCP-1 | 0.39(0.22) | 0.4(0.23) | 0.39(0.23) | 0.62 | 0.733 |
| Eotaxin | 0.52(0.2) | 0.51(0.22) | 0.52(0.21) | 0.97 | 0.615 |
| IL-17 | 0.54(0.22) | 0.54(0.22) | 0.54(0.23) | 0.25 | 0.882 |
| IL-4 | 0.44(0.27) | 0.45(0.25) | 0.46(0.29) | 0.14 | 0.931 |
| IL-2Rα | 0.5(0.25) | 0.5(0.24) | 0.5(0.25) | 0.40 | 0.820 |
| MIG | 0.29(0.28) | 0.3(0.24) | 0.29(0.23) | 2.92 | 0.233 |
| MIP-1β | 0.46(0.23) | 0.46(0.24) | 0.48(0.24) | 0.07 | 0.966 |
| IFN-α2 | 0.53(0.25) | 0.54(0.24) | 0.52(0.26) | 0.07 | 0.967 |
| SDF-1α | 0.48(0.21) | 0.47(0.23) | 0.48(0.23) | 0.03 | 0.986 |
| IL-16 | 0.24(0.23) | 0.23(0.24) | 0.24(0.22) | 1.44 | 0.486 |
| LIF | 0.41(0.26) | 0.41(0.25) | 0.4(0.25) | 0.38 | 0.828 |
| TNF-β | 0.72(0.24) | 0.7(0.24) | 0.71(0.25) | 1.40 | 0.496 |
| MIF | 0.33(0.25) | 0.34(0.25) | 0.32(0.21) | 1.08 | 0.584 |
| RANTES | 0.45(0.23) | 0.45(0.22) | 0.44(0.23) | 0.45 | 0.797 |
| IL-18 | 0.35(0.23) | 0.36(0.22) | 0.35(0.22) | 1.74 | 0.420 |
| PDGF-β | 0.37(0.26) | 0.36(0.25) | 0.38(0.25) | 0.00 | 0.999 |
| IP-10 | 0.29(0.23) | 0.29(0.25) | 0.29(0.22) | 0.39 | 0.824 |
| IL-13 | 0.42(0.24) | 0.42(0.25) | 0.42(0.25) | 0.03 | 0.986 |
| MIP-1α | 0.41(0.28) | 0.43(0.27) | 0.42(0.25) | 0.51 | 0.773 |
| G-CSF | 0.4(0.24) | 0.42(0.24) | 0.41(0.23) | 0.76 | 0.682 |
| GROα | 0.66(0.28) | 0.63(0.25) | 0.66(0.28) | 1.79 | 0.409 |
| HGF | 0.43(0.27) | 0.44(0.26) | 0.45(0.27) | 0.34 | 0.845 |
| IL-1α | 0.53(0.24) | 0.52(0.24) | 0.52(0.23) | 1.28 | 0.527 |
| SCF | 0.44(0.26) | 0.44(0.25) | 0.44(0.23) | 0.16 | 0.921 |
| TRAIL | 0.41(0.24) | 0.41(0.22) | 0.42(0.25) | 0.18 | 0.914 |
| M-CSF | 0.36(0.21) | 0.37(0.21) | 0.35(0.21) | 2.32 | 0.314 |
| CTACK | 0.48(0.24) | 0.47(0.24) | 0.47(0.25) | 0.08 | 0.960 |
| IL-7 | 0.5(0.26) | 0.5(0.26) | 0.52(0.27) | 0.50 | 0.780 |
| IL-9 | 0.43(0.25) | 0.43(0.24) | 0.44(0.24) | 0.07 | 0.967 |
| SCGF-β | 0.59(0.23) | 0.58(0.23) | 0.58(0.22) | 0.67 | 0.715 |

^1^ Data [i.e., mean (SD)] were first normalized and second corrected for batch effect.

**Table S6. The change levels of cytokines in three data sets.**

| Cytokines | Discovery Set  (n=37) | | | Validation Set  (n=42) | | | Control Set  (n=37) | | | $\boldsymbol{\chi}_{\boldsymbol{2}}^{\boldsymbol{2}}$ | P value ^4^ | |
| --- | --- | --- | --- | --- | --- | --- | --- | --- | --- | --- | --- | --- |
|  | **mean (SD)** | **t-stat ^1^** | **p-value** | **mean (SD)** | **t-stat ^2^** | **p-value** | **mean (SD)** | **t-stat ^3^** | **p-value** |  |  |  |
| IL-1β | 0.04(0.82) | 0.26 | 0.793 | 0.23(1.81) | 0.81 | 0.423 | -0.2(0.82) | -1.47 | 0.151 | 4.37 | | 0.218 |
| IL-6 | 0.47(1.19) | 2.41 | 0.021 | 0.96(2.08) | 2.99 | 0.005 | -0.27(1.26) | -1.31 | 0.200 | 11.47 | | 0.016 |
| IL-8 | 0.95(5.96) | 0.97 | 0.337 | 6.75(8.67) | 5.04 | 0.000 | 0.07(51.61) | 0.01 | 0.994 | 13.01 | | 0.010 |
| IFN-γ | -1.44(9.46) | -0.92 | 0.362 | -9.11(12.95) | -4.56 | 0.000 | -5.84(19.11) | -1.83 | 0.075 | 7.87 | | 0.060 |
| TNF-α | -0.01(22.99) | 0.00 | 0.998 | 0.04(15.99) | 0.01 | 0.988 | -1.63(13.91) | -0.72 | 0.476 | 0.54 | | 0.785 |
| MCP-1 | 10(25.88) | 2.32 | 0.026 | -7.48(14.12) | -3.43 | 0.001 | -0.28(5.4) | -0.32 | 0.753 | 13.86 | | 0.009 |
| Eotaxin | 6.39(38.31) | 1.01 | 0.317 | -28.03(26.5) | -6.85 | 0.000 | -2.43(12.21) | -1.23 | 0.228 | 27.11 | | 0.000 |
| IL-17 | 0.55(2.94) | 1.14 | 0.263 | 3.66(10.09) | 2.32 | 0.025 | -0.33(1.93) | -1.07 | 0.291 | 7.81 | | 0.060 |
| IL-4 | 0(1.91) | 0.01 | 0.994 | 0.09(2.03) | 0.28 | 0.779 | 0(0.31) | -0.08 | 0.935 | 0.86 | | 0.713 |
| IL-2Rα | -31.18(75.91) | -2.50 | 0.017 | -28.88(40.47) | -4.62 | 0.000 | -11.2(32.94) | -2.04 | 0.049 | 4.03 | | 0.245 |
| MIG | -89.67(940.12) | -0.57 | 0.571 | -49.97(278.71) | -1.16 | 0.252 | -60.84(248.79) | -1.49 | 0.146 | 1.57 | | 0.550 |
| MIP-1β | 3.32(25.68) | 0.79 | 0.436 | -3.51(17.49) | -1.30 | 0.201 | -3.23(18.3) | -1.07 | 0.290 | 2.54 | | 0.432 |
| IFN-α2 | 1.27(4.19) | 1.84 | 0.074 | 6.77(12.96) | 3.34 | 0.002 | 0.12(2.6) | 0.29 | 0.773 | 11.58 | | 0.016 |
| SDF-1α | 23.99(66.87) | 2.18 | 0.036 | -9.98(169.11) | -0.38 | 0.704 | -11.02(148.03) | -0.45 | 0.658 | 2.52 | | 0.432 |
| IL-16 | -12.94(93.01) | -0.82 | 0.416 | -62.63(91.32) | -4.44 | 0.000 | -0.29(25.02) | -0.07 | 0.945 | 17.79 | | 0.002 |
| LIF | -4.88(37.99) | -0.78 | 0.440 | 16.05(37.53) | 2.77 | 0.008 | -4.01(29.03) | -0.83 | 0.413 | 9.42 | | 0.035 |
| TNF-β | 6.23(29.53) | 1.28 | 0.208 | 3.93(26.26) | 0.97 | 0.338 | -4.89(28.1) | -1.06 | 0.297 | 1.74 | | 0.524 |
| MIF | 917.68(5881.86) | 0.91 | 0.370 | 107.19(452.17) | 1.54 | 0.132 | -25.05(323.71) | -0.48 | 0.636 | 4.50 | | 0.217 |
| RANTES | 679.43(7771.86) | 0.52 | 0.608 | -290.17(1433.64) | -1.31 | 0.197 | -108.11(1816.64) | -0.37 | 0.716 | 2.17 | | 0.493 |
| IL-18 | -12.66(74.67) | -1.03 | 0.309 | -10.91(21.1) | -3.35 | 0.002 | 0.07(51.61) | 0.01 | 0.994 | 2.00 | | 0.510 |

**Table S6 (continued).**

| PDGF-β | 221.3(1055.17) | 1.28 | 0.210 | 195.53(1322.14) | 0.96 | 0.343 | 20.17(249.99) | 0.50 | 0.622 | 3.62 | 0.287 |
| --- | --- | --- | --- | --- | --- | --- | --- | --- | --- | --- | --- |
| IP-10 | -94.53(184.66) | -3.11 | 0.004 | -160.17(337.53) | -3.08 | 0.004 | -55.07(183.03) | -1.83 | 0.075 | 1.83 | 0.519 |
| IL-13 | 0.43(2.34) | 1.13 | 0.267 | 0.73(3.49) | 1.36 | 0.181 | 0.21(1.25) | 1.02 | 0.316 | 1.41 | 0.557 |
| MIP-1α | 0.2(1) | 1.19 | 0.243 | 0.19(1) | 1.26 | 0.216 | -0.01(0.43) | -0.20 | 0.840 | 2.77 | 0.416 |
| G-CSF | -0.19(58.09) | -0.02 | 0.984 | 1.47(71.36) | 0.13 | 0.894 | 2.18(46.62) | 0.29 | 0.775 | 0.37 | 0.831 |
| GROα | 97.44(387.95) | 1.53 | 0.135 | 117.14(172.24) | 4.41 | 0.000 | -10.2(262.22) | -0.24 | 0.814 | 5.40 | 0.147 |
| HGF | 35.99(131.03) | 1.67 | 0.103 | -55.14(85.53) | -4.18 | 0.000 | -2.16(82.92) | -0.16 | 0.873 | 10.90 | 0.019 |
| IL-1α | 4(11.85) | 2.05 | 0.048 | 13.2(25.07) | 3.41 | 0.001 | 0.96(16.36) | 0.36 | 0.722 | 5.59 | 0.143 |
| SCF | 2.62(16.64) | 0.96 | 0.345 | -6.45(18) | -2.32 | 0.025 | 1.96(17.44) | 0.68 | 0.498 | 6.42 | 0.101 |
| TRAIL | -5.02(21.32) | -1.43 | 0.161 | -2.21(18.52) | -0.77 | 0.445 | 0.15(13.64) | 0.07 | 0.947 | 0.58 | 0.785 |
| M-CSF | -1.24(8.34) | -0.90 | 0.372 | -7.84(14.19) | -3.58 | 0.001 | 0.69(28.53) | 0.15 | 0.885 | 6.54 | 0.101 |
| CTACK | -6.42(234.53) | -0.17 | 0.869 | -125.41(132.2) | -6.15 | 0.000 | -45.15(159.97) | -1.69 | 0.099 | 16.18 | 0.004 |
| IL-7 | 3.19(8.37) | 2.31 | 0.026 | 6.5(19.6) | 2.10 | 0.042 | 0.15(2.6) | 0.35 | 0.725 | 7.77 | 0.060 |
| IL-9 | 2.33(66.51) | 0.21 | 0.835 | 9.71(29.44) | 2.14 | 0.039 | -2.48(50.44) | -0.30 | 0.764 | 1.94 | 0.510 |
| SCGF-β | 3904.43(17950.5) | 1.32 | 0.194 | -1350.1(28633.11) | -0.31 | 0.761 | -1442.32(18302.91) | -0.48 | 0.635 | 1.41 | 0.557 |

^1^ The degree of freedom for the One sample t-test statistic is 36.

^2^ The degree of freedom for the One sample t-test statistic is 41.

^3^ The degree of freedom for the One sample t-test statistic is 36.

^4^ FDR adjustment for multiple testing.

**Table S7. Correlation between the identified canonical components after controlling for baseline variables.**

Sensitivity analysis was conducted by re-evaluating this correlation after controlling for potential confounders at the baseline.

|  | **tval^1^** | **P value** |
| --- | --- | --- |
| **MIG** | 2.14 | 0.039^*^ |
| **IFN-α2** | 2.06 | 0.046^*^ |
| **IFN-γ** | 2.33 | 0.025^*^ |
| **CARS_S** | 2.27 | 0.029^*^ |
| **CARS_N** | 2.22 | 0.032^*^ |
| **CARS_D** | 2.28 | 0.028^*^ |
| **gender** | 2.15 | 0.038^*^ |
| **age** | 2.26 | 0.029^*^ |
| **BMI** | 2.25 | 0.030^*^ |

^1^ The degree of freedom for the Student's t-test statistic is 37.

^*^ P value is less than 0.05.

CARS_S, CARS score on social impairment domain; CARS_N, CARS score on negative emotionality domain; CARS_D, CARS score on distorted sensory response domain; IFN-**γ**, Interferon gamma; IFN-α2, Interferon alpha 2; MIG, Monokine induced by gamma interferon; BMI, Body Mass Index.

**Table S8. Comparison of AUC between the models including and without including the cytokines to predict the immuno-behaviourally defined best responding group and the least responding group.**

|  | **Best responding group** | | **Least responding group** | |
| --- | --- | --- | --- | --- |
| **Cytokines** | **included** | **not included** | **included** | **not included** |
| **ORF** | 0.747 | 0.632 | 0.712 | 0.520 |
| **SVM** | 0.726 | 0.614 | 0.681 | 0.510 |
| **PLS** | 0.797 | 0.655 | 0.680 | 0.772 |
| **sLDA** | 0.832 | 0.772 | 0.688 | 0.772 |
| **NN** | 0.739 | 0.557 | 0.729 | 0.516 |

AUC, area under ROC; ORF – Oblique Random Forest; SVM – support vector machine; PLS – Partial Least Squares, sLDA – sparse Linear Discriminant Analysis, NN – Neural Networks

**Table S9. Comparison of AUC between the models predicting the immuno-behaviourally defined responder group and the behaviourally defined responder group**

|  | **Mean AUC(SD)** | | **Mean**  **ΔAUC(SD)^1^** | **95% CI^1^** |
| --- | --- | --- | --- | --- |
|  | **immuno-behaviourally defined the best responding group** | **behaviourally defined responder group** |  |  |
| **ORF** | 0.747(0.103) | 0.643(0.076) | 0.154(0.149) | (0.125, 0.184) |
| **sLDA** | 0.832(0.041) | 0.529(0.095) | 0.303(0.081) | (0.287, 0.319) |
| **NN** | 0.739(0.123) | 0.489(0.078) | 0.250(0.134) | (0.224, 0.277) |
| **PLS** | 0.797(0.106) | 0.566(0.099) | 0.181(0.151) | (0.151, 0.211) |
| **SVM** | 0.726(0.124) | 0.616(0.091) | 0.110(0.165) | (0.077, 0.142) |

^1^ Models was constructed by 100 bootstraps.

AUC, area under ROC; CI, confidence interval; SD, standard deviation; ORF, Oblique Random Forest; PLS, Partial Least Squares; SVM, Support Vector Machine; sLDA, sparse Linear Discriminant Analysis; NN, Neural Networks; ΔAUC, the differences between AUC of models with and without each cytokine.

**Figure S1. Adjustment for batch effects.**

PCA plots show the impact of ‘ComBat’ algorithm adjustment on batch effect in three batches. Both before and after ComBat adjustment, scatter plots of the first two principal components are displayed (PC1 vs PC2). In these plots, individual patient samples are represented by dots and color-coded according to their batch of origin. Post-ComBat adjustment the batches demonstrated more homogeneity, as evidenced by increased overlap in PCA scatter plots.


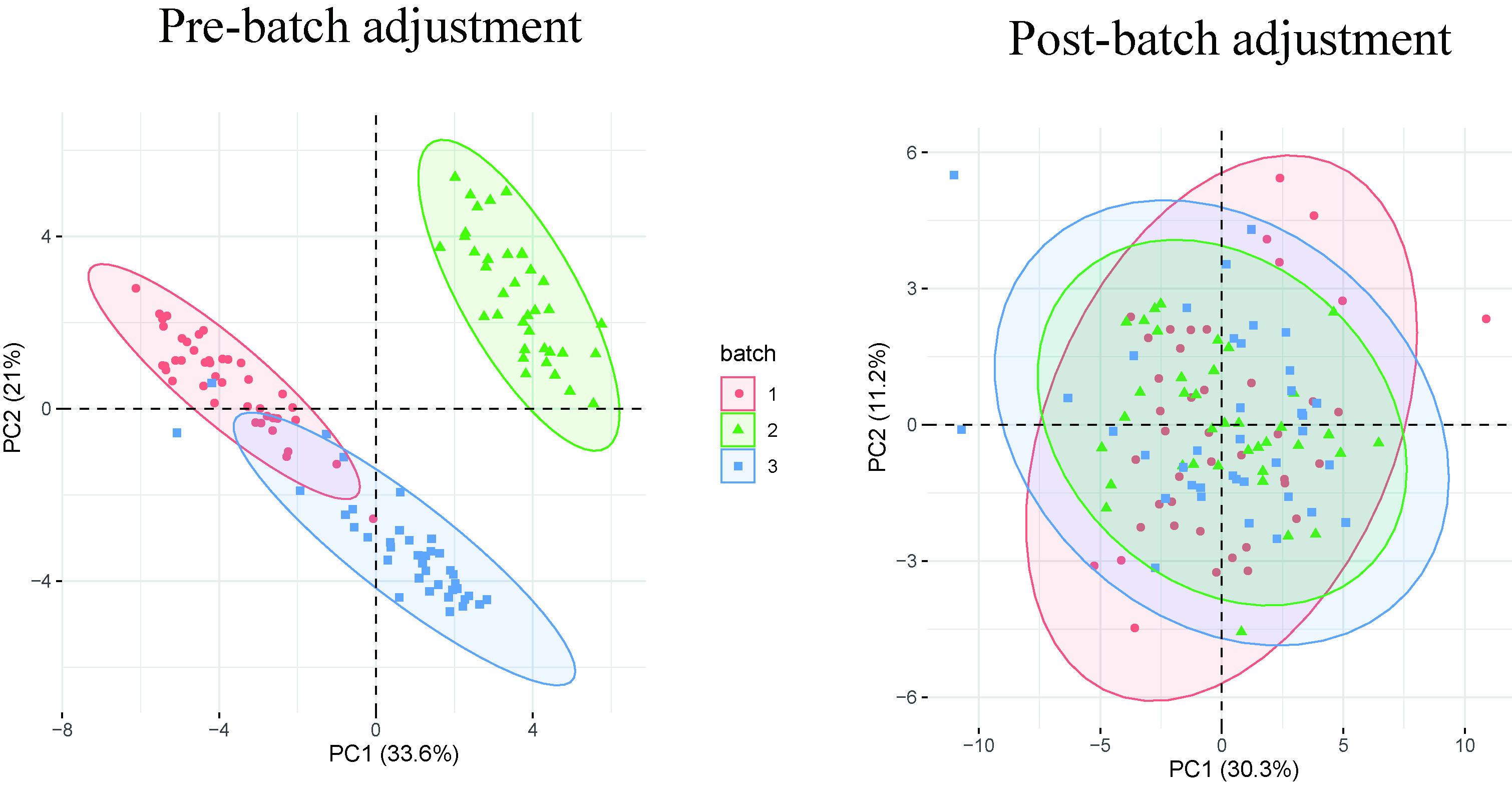


**Figure S2. Pairwise association between CARS total score and cytokine.**

Partial correlation heatmaps are showed for pairwise association between **[A]** the baseline CARS total score and the baseline cytokine levels, **[B]** the baseline CARS total score and the changes of cytokine levels, **[C]** the change of CARS total score and the baseline cytokine levels, and **[D]** the change of CARS total score and the changes of cytokine levels. The associations were carried out FDR adjustment.


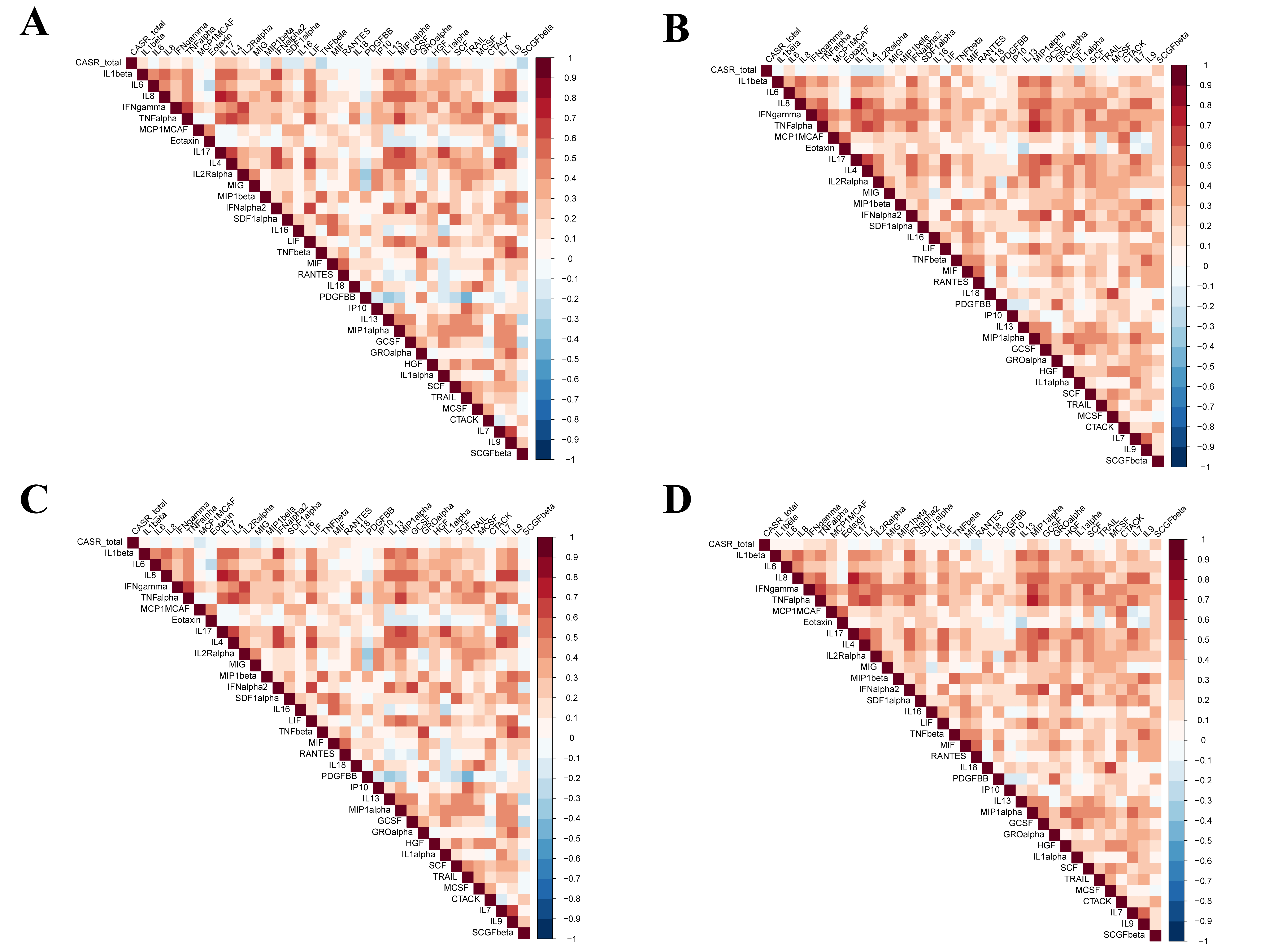


**Figure S3. The scree plot for selection of the optimal number of clusters.**

We generated different clusters, with the number of clusters (k) ranging from 2 to 14. To validate clustering outcomes, we used internal cluster quality measures. For each of the possible number of clusters, an index is calculated reflecting the between-subject similarity within clusters and the dissimilarity between clusters. This index usually increases monotonically with increasing number of clusters, and the optimal value is determined to be at the elbow of its plot, where the change in index (difference with k-1 and k+1) is at a maximum. There are several possible indices available. We used the cluster number most of them chose. The following plots show the Hubert statistic for k of 2 to 14 possible clusters and the change (delta) in index compared with k-1. The results from the internal cluster quality indexes produce a good signal at 3 clusters.


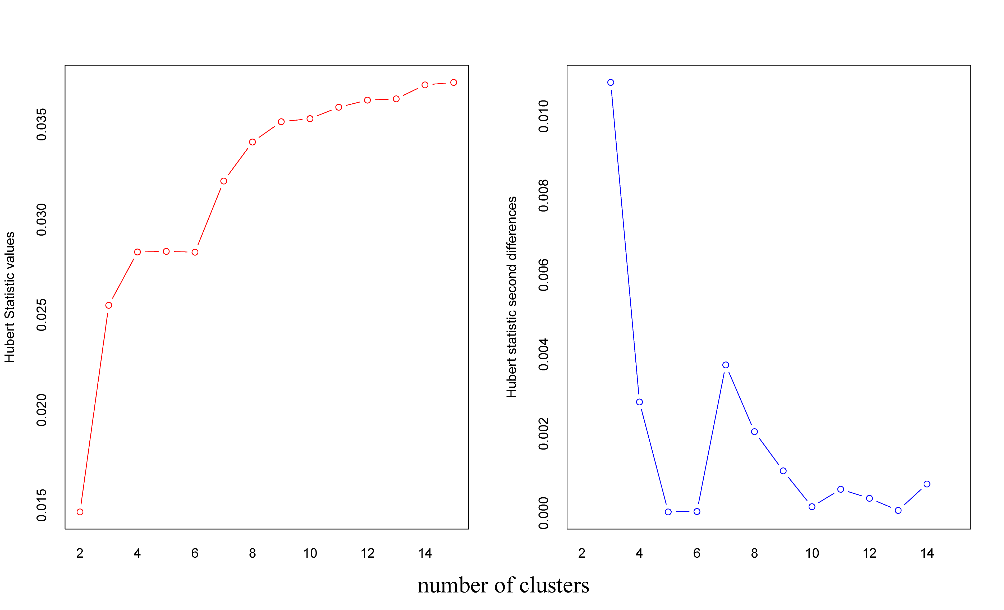


**Figure S4. Boxplot for the changes of CARS and cytokine levels in 3 immuno-behavioural groups.**


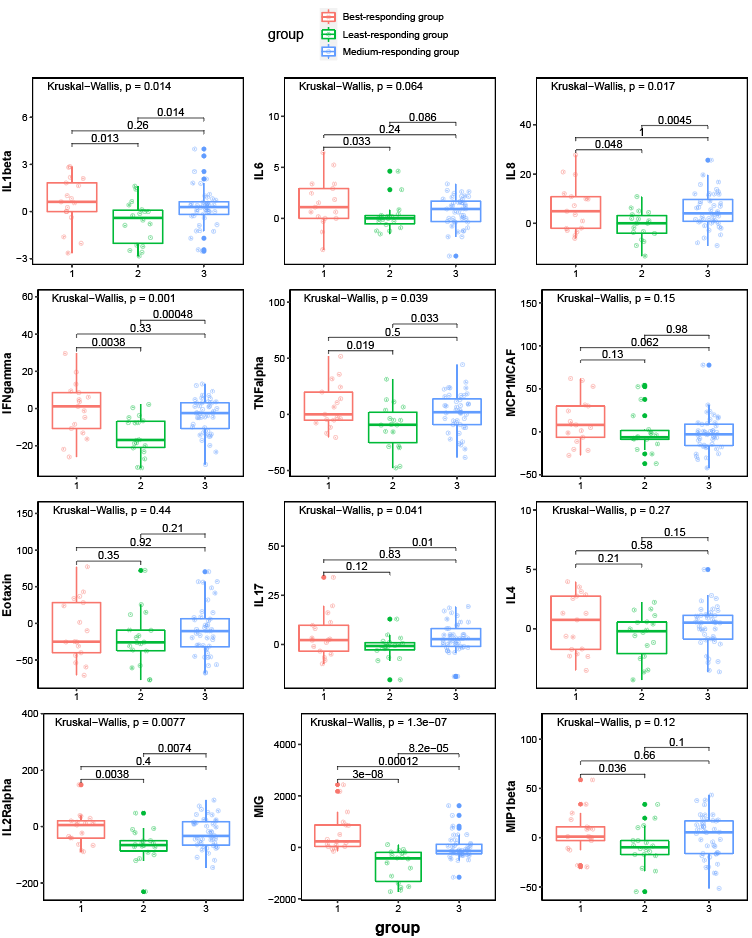


**Figure S4 (continued).**


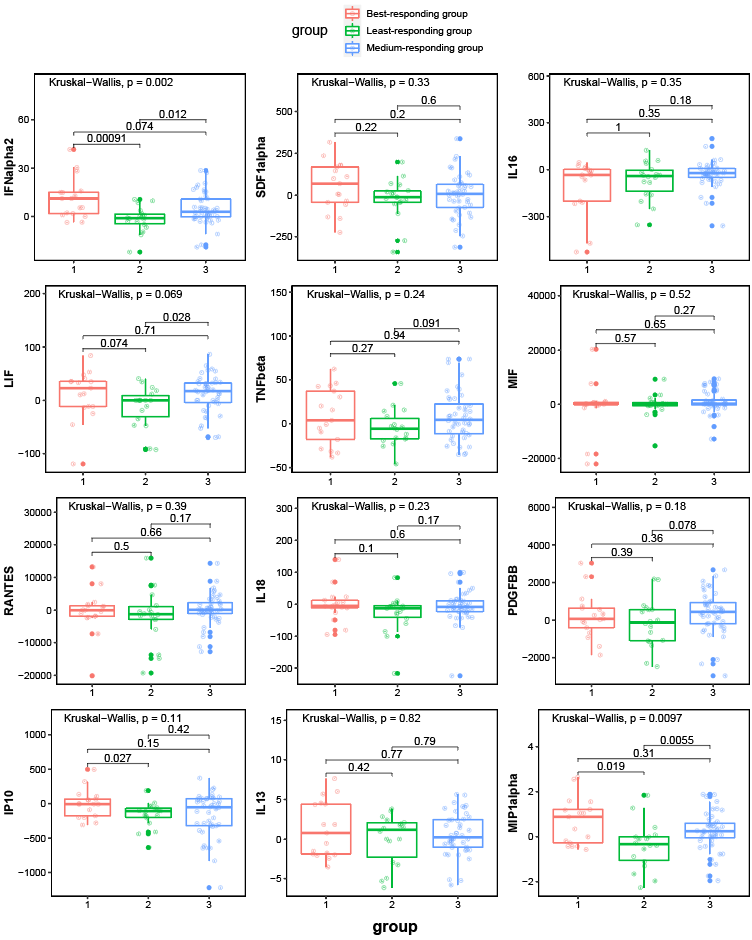


**Figure S4 (continued).**


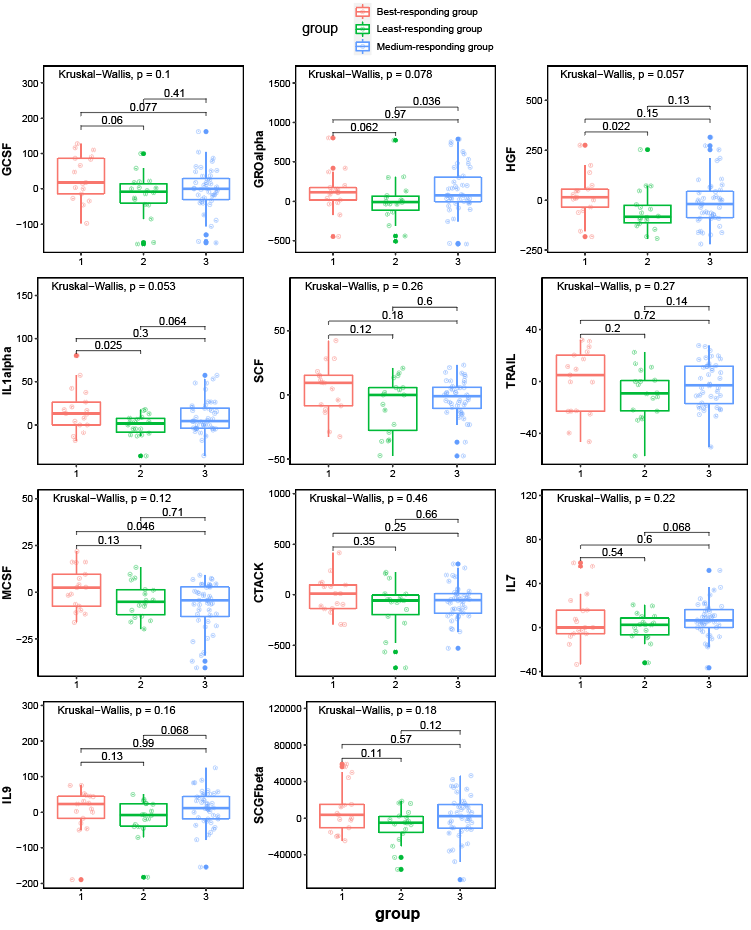


**Figure S5. ROC curve for the prediction of treatment response defined by CARS.**

**
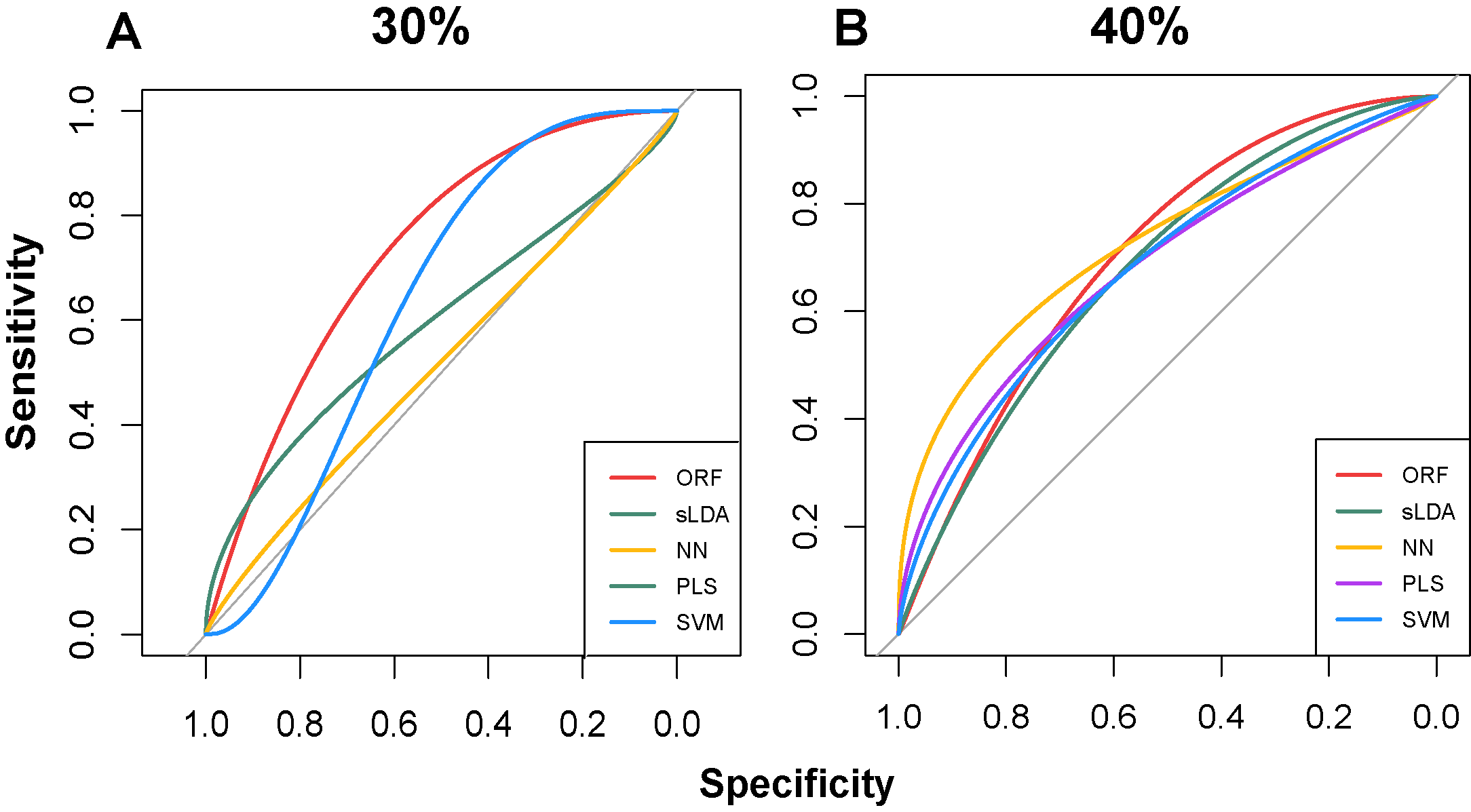
**

The classifiers included the Oblique Random Forest (ORF) model, Partial Least Squares (PLS) model, Support Vector Machine (SVM) model, sparse Linear Discriminant Analysis (sLDA) model and Neural Networks (NN) model. Based on the behavioural assessments at the baseline before treatment, the models were trained to predict the response to bumetanide for the children with ASD. The responders were identified as their CARS total score decreased greater than 2.5 points(A) or 2 points(B) after a 3-month treatment of bumetanide. As described in the main text, the models were trained using the Discovery Set, and tested using the Validation Set. The performances of the classification accuracy in the test data set were reported in this figure.
